# Extensive hydrogen incorporation is not necessary for superconductivity in topotactically reduced nickelates

**DOI:** 10.1038/s41467-024-51479-3

**Published:** 2024-08-27

**Authors:** Purnima P. Balakrishnan, Dan Ferenc Segedin, Lin Er Chow, Paige E. Quarterman, Shin Muramoto, Mythili Surendran, Ranjan K. Patel, Harrison LaBollita, Grace A. Pan, Qi Song, Yang Zhang, Ismail El Baggari, Koushik Jagadish, Yu-Tsun Shao, Berit H. Goodge, Lena F. Kourkoutis, Srimanta Middey, Antia S. Botana, Jayakanth Ravichandran, A. Ariando, Julia A. Mundy, Alexander J. Grutter

**Affiliations:** 1 NIST Center for Neutron Research, National Institute of Standards and Technology, Gaithersburg, MD 20899 USA; 2 Department of Physics, Harvard University, Cambridge, MA 02138 USA; 3 Department of Physics, Faculty of Science, National University of Singapore, Singapore, 117551 Singapore; 4 Material Measurement Laboratory, National Institute of Standards and Technology, Gaithersburg, MD 20899 USA; 5 Mork Family Department of Chemical Engineering and Materials Science, University of Southern California, Los Angeles, CA 90089 USA; 6 Core Center for Excellence in Nano Imaging, University of Southern California, Los Angeles, CA 90089 USA; 7 Department of Physics, Indian Institute of Science, Bengaluru, 560012 India; 8 Department of Physics, Arizona State University, Tempe, AZ 85287 USA; 9 The Rowland Institute at Harvard, Harvard University, Cambridge, MA 02138 USA; 10 Core Center for Excellence in Nano Imaging, University of Southern California, 925 Bloom Walk, Los Angeles, CA 90089 USA; 11 School of Applied and Engineering Physics, Cornell University, Ithaca, NY 14853 USA; 12 Kavli Institute at Cornell for Nanoscale Science, Ithaca, NY 14853 USA; 13 Max Planck Institute for Chemical Physics of Solids, 01187 Dresden, Germany; 14 Ming Hsieh Department of Electrical and Computer Engineering, University of Southern California, Los Angeles, CA 90089 USA

**Keywords:** Superconducting properties and materials, Surfaces, interfaces and thin films

## Abstract

A key open question in the study of layered superconducting nickelate films is the role that hydrogen incorporation into the lattice plays in the appearance of the superconducting state. Due to the challenges of stabilizing highly crystalline square planar nickelate films, films are prepared by the deposition of a more stable parent compound which is then transformed into the target phase *via* a topotactic reaction with a strongly reducing agent such as CaH_2_. Recent studies, both experimental and theoretical, have introduced the possibility that the incorporation of hydrogen from the reducing agent into the nickelate lattice may be critical for the superconductivity. In this work, we use secondary ion mass spectrometry to examine superconducting La_1−*x*_*X*_*x*_NiO_2_ / SrTiO_3_ (*X* = Ca and Sr) and Nd_6_Ni_5_O_12_ / NdGaO_3_ films, along with non-superconducting NdNiO_2_ / SrTiO_3_ and (Nd,Sr)NiO_2_ / SrTiO_3_. We find no evidence for extensive hydrogen incorporation across a broad range of samples, including both superconducting and non-superconducting films. Theoretical calculations indicate that hydrogen incorporation is broadly energetically unfavorable in these systems, supporting our conclusion that extensive hydrogen incorporation is not generally required to achieve a superconducting state in layered square-planar nickelates.

## Abstract (web summary)

The role of hydrogen in engendering superconductivity in layered nickelates is under intense debate. Here, the authors perform secondary ion mass spectroscopy and see no evidence for extensive hydrogen incorporation into superconducting nickelates.

## Introduction

**Fig. 1:**
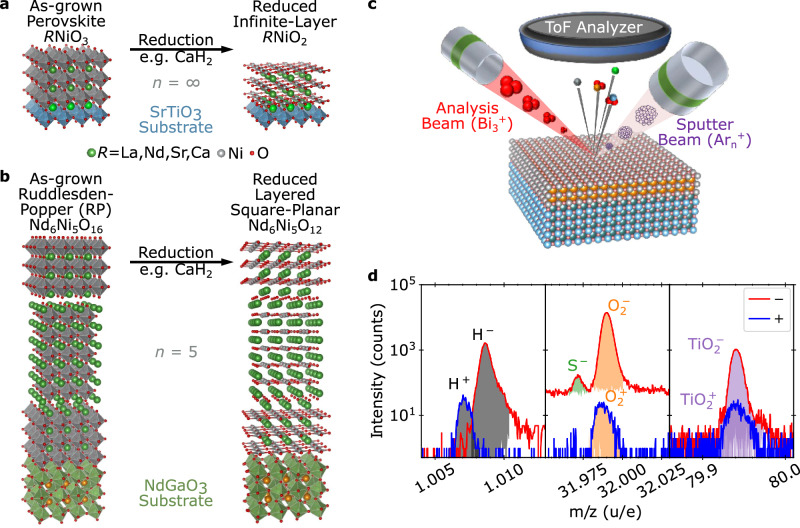
Representation of materials and methods used in this study. Schematic crystal structures for precursor phase and reduced **a** *n* = *∞* and **b** *n* = 5 layered square-planar nickelate compounds. **c** Schematic of the ToF-SIMS measurement technique. **d** SIMS spectra measured separately for positive and negative ions are analyzed by identifying peaks by mass-to-charge ratio (*m*/*z*) and extracting integrated area.

Superconductivity in nickelates has been pursued ever since the discovery of the cuprates^1–5^, but it was not until 2019 that it was demonstrated in thin films of the infinite-layer compound NdNiO_2_ via hole doping with Sr^6^. This discovery introduced a novel family of layered nickelate superconductors that has now been extended to include the Pr- and La- analogs of the infinite-layer compound as well as the five-layer material Nd_6_Ni_5_O_12_^7–12^. While superconducting nickelates exhibit many interesting phenomena^13–17^, they also represent a unique materials synthesis challenge^18–22^. In general, layered square-planar nickelates cannot be synthesized directly, instead requiring a two-step fabrication method wherein an oxygen-rich precursor material is grown by traditional thin film deposition methods and then topotactically reduced, as illustrated in Fig. 1a, b^23^. Typically, the reduction is performed *via* a thermal anneal employing a chemical reducing agent and oxygen sink, such as H_2_, NaH, or CaH_2_^6,24–26^. One of the most pressing open questions is the degree to which the reduction process incorporates hydrogen into the nickelate film, and whether hydrogen is important in stabilizing superconductivity.

A notable recent study by Ding et al. reported that hydrogen is critical for the emergence of superconductivity, requiring a stoichiometry around Nd_0.8_Sr_0.2_NiO_2_H_0.25_^27^. However, in this study, hydrogen and oxygen stoichiometry are highly correlated through reduction. Furthermore, recent results using alternative reduction processes with no hydrogen source have cast doubt on the role of incorporated hydrogen on the electronic state^9,25^. Previous theoretical works have argued that *R*NiO_2_ (*R* = La, Nd) could be energetically unstable with respect to topotactic hydrogen, significantly altering the electronic structure^28–30^. In light of Ref. ^27^, some calculations have shown that an optimal H concentration may be beneficial to promote superconductivity^31^, while others have indicated that the electron phonon-coupling in hydrogen-intercalated nickelates is not strong enough to drive electron pairing and thus cannot be responsible for the superconductivity^32^.

Given the differing conclusions in the literature, a comprehensive examination of the role of hydrogen incorporation in superconducting nickelates is urgently needed. To understand more broadly applicable trends rather than the specifics of one sample type or fabrication protocol, we used time-of-flight secondary ion mass spectrometry (ToF-SIMS) to study the relationship between hydrogen incorporation and superconductivity in a broad range of nickelate films grown and reduced by three different research groups. The films used in this study were grown *via* either molecular beam epitaxy (MBE) or pulsed laser deposition (PLD), in a variety of geometries, and reduced with CaH_2_ at different conditions. As the energetics of incorporating hydrogen may vary greatly depending on stoichiometry and structure^28^, we compared multiple nickelate systems, including superconducting examples of La_1−*x*_Ca_*x*_NiO_2_, La_1−*x*_Sr_*x*_NiO_2_, and Nd_6_Ni_5_O_12_. We also examined non-superconducting NdNiO_2_ and Nd_1−*x*_Sr_*x*_NiO_2_. We find no evidence that a large concentration of incorporated hydrogen is necessary to observe superconductivity. Instead, a wide range of films, superconducting and non-superconducting, exhibit H^−^ intensities that are similar to the substrate background. Theoretical calculations support this picture, revealing that hydrogen incorporation is energetically unfavorable across all materials studied in this work.

## Results

As illustrated in Fig. 1c, ToF-SIMS is a destructive technique in which an ion beam sputters through the film, and ejected molecular ions are analyzed using a mass spectrometer to provide a depth- and element-resolved picture of the ejected species and, thereby, chemical composition. ToF-SIMS allows the isolation and identification of elemental H^±^, and O^±^ as well as larger ejected molecules such as OH^±^, ${{{{\rm{O}}}}}_{2}^{\pm }$, and ${{{{\rm{TiO}}}}}_{2}^{\pm }$, as shown in Fig. 1d. The change in molecular species intensity over time as the sample is sputtered results in a depth-resolved understanding of the chemical composition with depth, in which layers only a few nm thick may be readily separated.

However, the measured intensity depends significantly on the sputtering conditions, chemical environment, film composition, density, electronic state, and prevalence of structural defects. Absolute scaling of stoichiometry and depth, therefore, requires calibration standards with known stoichiometry and a similar chemical environment to the films of interest. Since the defect levels and chemistry in superconducting nickelates evolve extensively during the reduction process, such standards are nearly impossible to obtain, and we instead adopt the convention of Ding et al., in which the hydrogen level observed in the SrTiO_3_ or NdGaO_3_ substrate is considered to be the background level representing minimal hydrogen^27^.

### Superconducting La_1−*x*_(Sr,Ca)_*x*_NiO_2_

**Fig. 2:**
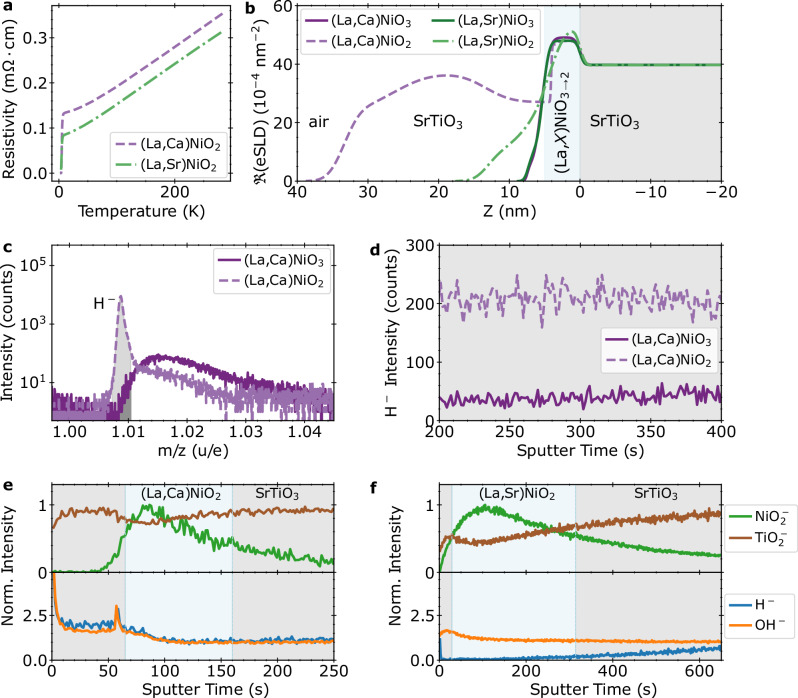
Characterization of superconducting La_*x*_(Sr,Ca)_1−*x*_NiO_2_ films. **a** Resistivity vs. temperature for the superconducting La_0.78_Ca_0.22_NiO_2_ and La_0.8_Sr_0.2_NiO_2_ samples, showing a clear transition and large RRR. **b** XRR depth profiles of the as-grown and superconducting films, specifically the real component of the scattering length density (SLD). **c** Intensity vs. mass-to-charge ratio near the H^−^ peak for an as-grown La_0.78_Ca_0.22_NiO_3_ and superconducting La_0.78_Ca_0.22_NiO_2_ film, integrated over the entire measurement time. **d** Raw intensity (counts) of the H^−^ peak in the substrate region for the same as-grown La_0.78_Ca_0.22_NiO_3_ and superconducting La_0.78_Ca_0.22_NiO_2_. **e** SIMS depth profile of superconducting La_0.78_Ca_0.22_NiO_2_. **f** SIMS depth profile of superconducting La_0.8_Sr_0.2_NiO_2_. Note that the La_0.8_Sr_0.2_NiO_2_ film was sputtered at a lower ion beam energy than the La_0.78_Ca_0.22_NiO_2_ due to the thinner cap layer.

We first present results from two doped superconducting infinite-layer systems: La_0.78_Ca_0.22_NiO_2_ and La_0.8_Sr_0.2_NiO_2_ grown by pulsed laser deposition on SrTiO_3_ substrates, as described in the Methods section. The quality of representative samples has been previously demonstrated through X-ray diffraction (XRD), cross-sectional scanning transmission electron microscopy (STEM), and electron energy loss spectroscopy (EELS) analysis^33,34^. To ensure depth-wise uniformity, the film thickness was limited to below 6 nm. Figure 2a shows the superconducting transitions for these samples, which have a residual resistivity ratio  ≈4.1, comparable to the highest reported values so far^21,35^. After reduction, an amorphous SrTiO_3_ cap is deposited to act as an oxidation barrier, with varying thickness due to the challenges associated with room-temperature growth. Film and cap thicknesses were verified using X-ray reflectometry (XRR), shown in Fig. 2b, which reveals that the initial perovskite phases are uniform with the expected scattering length densities. After reduction, the sharp interfaces slightly roughen, likely linked to the energetic deposition of the caps. While here we focus on superconducting films, we also measured the as-grown perovskite film from the same growth, the details of which can be found in the Supplementary Information.

To understand the sensitivity of this experiment to hydrogen, we first compare the overall hydrogen content of as-grown La_0.78_Ca_0.22_NiO_3_ and superconducting La_0.78_Ca_0.22_NiO_2_ samples in Fig. 2c. Here, we show the H^−^ peak for both samples integrated across all sputtering times. The as-grown sample contains negligible hydrogen within either the film or substrate, indicating an extremely clean growth and handling process. The superconducting sample, in contrast, exhibits a clearly-resolved H^−^ peak much larger than the measurement background. The additional hydrogen introduced into the reduced superconducting sample is easily detectable. We next compare the integrated peak intensity, indicated by the shaded region, over sputter time (depth). For this same pair of samples, Fig. 2d shows the H^−^ intensity in just the SrTiO_3_ substrate. Interestingly, while the substrate intensity of the as-grown sample is less than 40 counts per frame, the SrTiO_3_ substrate associated with the superconducting La_0.78_Ca_0.22_NiO_2_ is an excellent match for the samples in Ding et al.^27^, with approximately 200 counts per frame of H^−^. Thus we may be confident that the observed hydrogen levels in the substrates are above the instrumental detection limit and closely match previous observations.

Having firmly established that the hydrogen levels reported previously in superconducting films are readily detectable with the instrument used in this study, we show SIMS data from superconducting La_0.78_Ca_0.22_NiO_2_ and La_0.8_Sr_0.2_NiO_2_ in Fig. 2e, f. Here the ${{{{\rm{NiO}}}}}_{2}^{-}$ and ${{{{\rm{TiO}}}}}_{2}^{-}$ intensities are normalized to their maximum values while the H^−^ and OH^−^ intensities are normalized to the steady-state value within the substrate; alternative normalizations and raw counts are shown in Supplementary Note 5. The film and substrate positions are indicated by the peak and dip in ${{{{\rm{NiO}}}}}_{2}^{-}$ and ${{{{\rm{TiO}}}}}_{2}^{-}$ intensities, respectively. The trends in H^−^ and OH^−^ intensities clearly disagree with prior reports: the superconducting La_0.78_Ca_0.22_NiO_2_ and La_0.8_Sr_0.2_NiO_2_ films do not exhibit the large 1–3 order of magnitude increases in H^−^ or OH^−^ signal which would be expected for extensive, multiple-percent hydrogen incorporation^27,36–38^.

Instead, apart from the quickly decaying signal associated with surface adsorbates, the H^−^ and OH^−^ signals within the La_0.78_Ca_0.22_NiO_2_ film are invariant within a factor of two of the signals within the substrate. Interestingly, the La_0.78_Ca_0.22_NiO_2_ sample, with a thicker amorphous SrTiO_3_ cap (29 nm), exhibits higher H^−^ intensity within the cap than within the nickelate film, concentrated near the interface. In the La_0.8_Sr_0.2_NiO_2_ sample with a thinner SrTiO_3_ cap (approximately 6 nm), H^−^ is much lower in the nickelate film than either the SrTiO_3_ substrate or the other superconducting film. We speculate that the SrTiO_3_ cap may play a role in hydrogen capture or transport^39^. Most importantly, the coexistence of different low hydrogen concentrations with superconductivity definitively demonstrates that extensive hydrogen doping is not required for superconductivity in the infinite-layer nickelates.

### Superconducting Nd_6_Ni_5_O_12_

**Fig. 3:**
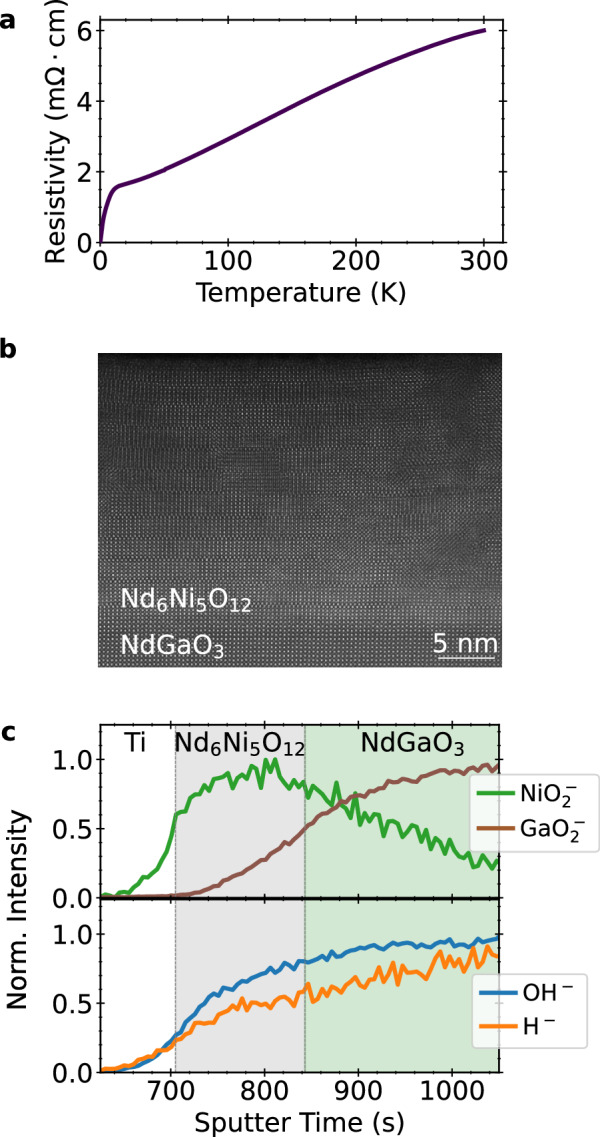
Characterization of a superconducting Nd_6_Ni_5_O_12_ film. **a** Temperature-dependent resistivity of reduced Nd_6_Ni_5_O_12_/NdGaO_3_ showing a clear superconducting transition. The same data as in Ref. ^10^. **b** STEM image of the reduced, superconducting Nd_6_Ni_5_O_12_ film and NdGaO_3_ substrate. **c** SIMS depth profiles of superconducting Nd_6_Ni_5_O_12_.

To test whether our findings are applicable more broadly within the square-planar nickelate family, beyond the infinite-layer structure, we examine the superconducting quintuple-layer nickelate Nd_6_Ni_5_O_12_. This film consists of 23 nm Nd_6_Ni_5_O_12_ synthesized on NdGaO_3_ (110) (see Synthesis Section 2 for details), with 10 nm titanium followed by 100 nm platinum patterned on the film surface as electrodes. Figure 3a shows the zero-field superconducting transition of this sample from Ref. ^10^, with a residual resistivity ratio of 3.8. Further characterization of this sample can be found in Ref. ^10^. Figure 3b shows a representative STEM image of this sample, revealing the five-layer square-planar structure.

Figure 3c plots the SIMS depth profile of this superconducting sample. ${{{{\rm{NiO}}}}}_{2}^{-}$ and ${{{{\rm{GaO}}}}}_{2}^{-}$ peaks are normalized to their maximum values, and clearly identify the electrode, film, and substrate regions. As before, we obtain information regarding the hydrogen concentration by examining the relative intensity of the H^−^ and OH^−^ peaks in the film and substrate. An advantage of the relatively thick electrode is the removal of surface contaminant effects from the measurement. Both the H^−^ and OH^−^ intensities are low in the platinum and titanium, increase slowly in the Nd_6_Ni_5_O_12_ film, and further increase deeper into the NdGaO_3_ substrate. Similar to the superconducting La_0.8_Sr_0.2_NiO_2_ sample, we find that the hydrogen content appears to be highest in the substrate, although again the nickelate film and substrate intensities are very similar. Once again there is no evidence of an order of magnitude increase in H^−^ intensity in the film.

### Non-superconducting films

With little evidence of extensive hydrogen incorporation in high-quality superconducting samples, the question remains whether some structures or processes are more susceptible to hydrogen. We speculate that films with increased defect densities, whether due to growth conditions or from long or overly aggressive reduction treatments, may incorporate additional hydrogen as a defect compensation mechanism. These films do not exhibit superconductivity, but do provide a mechanism for understanding the extent to which hydrogen can be incorporated during reduction and whether it might inhibit the fabrication of superconducting films.

**Fig. 4:**
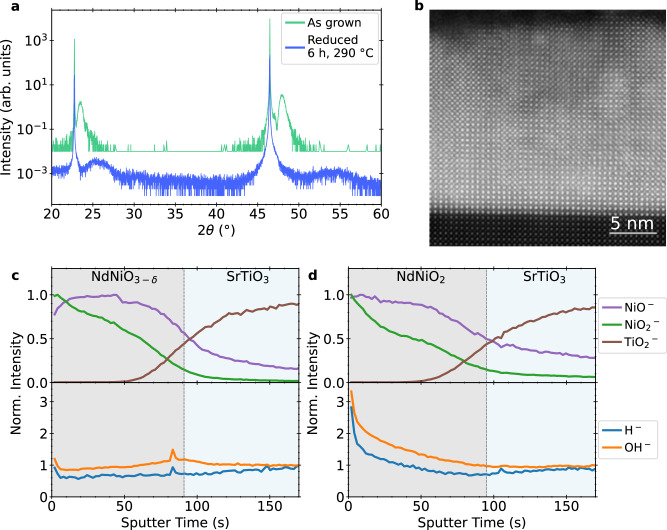
Characterization of non-superconducting NdNiO_2_ films. **a** XRD of as-grown NdNiO_3_ and reduced NdNiO_2_ films showing partial reduction to the infinite-layer phase. **b** STEM of an equivalent sister NdNiO_2_ sample revealing extended defects concentrated near the surface. **c** SIMS depth profile of the as-grown NdNiO_3_ film. **d** SIMS depth profile of the NdNiO_2_ film, reduced for an extended time of 6 h at 290 °C. SIMS shows increased hydrogen concentration at the surface even without full oxygen removal.

We first consider 17 nm NdNiO_3_/SrTiO_3_ (001) films grown by MBE and subjected to an incomplete reduction, at a lower temperature but for longer times compared to the optimized treatment for achieving high-quality NdNiO_2_. XRD scans shown in Fig. 4a indicate a reduction toward the infinite-layer NdNiO_2_ phase, but with a modest decrease in crystallinity. Electron microscopy measurements on an equivalent sister sample, shown in Fig. 4b, reveal the presence of defects and phase boundaries, as expected.

The film was cut in half before reduction, and both as-grown and reduced samples were measured using ToF-SIMS, yielding the intensity depth profiles in Fig. 4c, d. As before, the NiO^−^, ${{{{\rm{NiO}}}}}_{2}^{-}$, and ${{{{\rm{TiO}}}}}_{2}^{-}$ peaks are normalized to their maximum value while the H^−^ and OH^−^ are normalized to the steady-state values in the substrate. The H^−^ and OH^−^ signals are slightly higher in the SrTiO_3_ substrate than in the as-grown NdNiO_3_ film. Upon reduction, H^−^ and OH^−^ increase at the surface of the films, and the lineshape of this increase only partially matches that of various peaks including ${{{{\rm{C}}}}}_{2}^{-}$ and Ca^+^ (see Supplementary Note 3), indicating that they do not solely originate from surface adsorbates introduced during the reduction process. Near the substrate interface, which has previously been shown to be the highest-quality region of the film^20,33^, the H^−^ intensity remains lower than in the SrTiO_3_ substrate. Thus, while some insignificant hydrogen content may be introduced during the reduction process, it seems to be limited near the surface of these uncapped films.

We next present our findings on non-superconducting infinite-layer Nd_0.8_Sr_0.2_NiO_2_/SrTiO_3_ films, which are appropriately doped to result in superconductivity but were reduced aggressively at high temperatures (600 °C compared to  ~300 °C); this reduction is enough to significantly hydrogen-dope a similar perovskite material, BaZrO_3_^38^. Furthermore, a common practice is to cap perovskite nickelate films with SrTiO_3_ prior to reduction to provide balanced strain for structural stability of the film throughout its entire thickness^6,20^. We, therefore, compare samples with and without a SrTiO_3_ capping layer grown in situ on the 10 nm Nd_0.8_Sr_0.2_NiO_3_ before reduction.

**Fig. 5:**
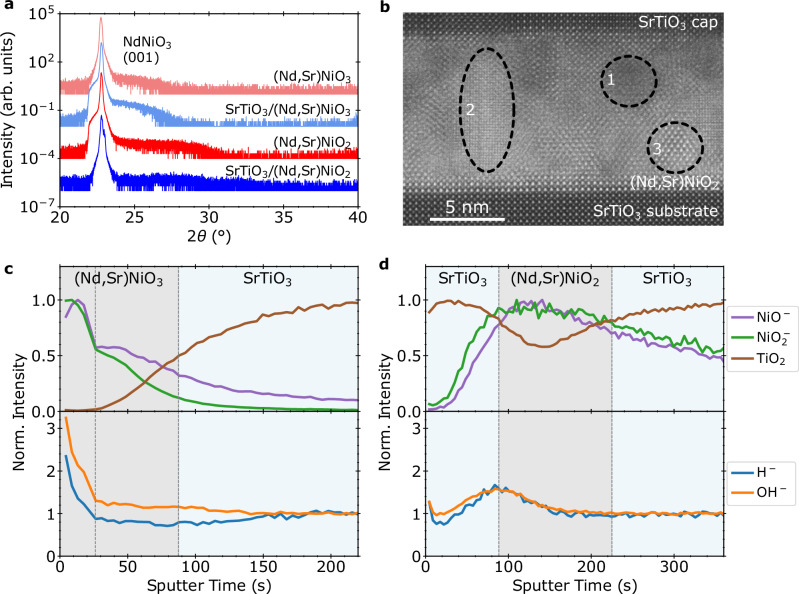
Characterization of Nd_*x*_Sr_1−*x*_NiO_3_ and non-superconducting Nd_*x*_Sr_1−*x*_NiO_2_ films. **a** XRD on Nd_0.8_Sr_0.2_NiO_3_ films grown by PLD, with and without a 10 nm SrTiO_3_ cap, showing a weak film (001) peak both before and after reduction, indicating a low-crystallinity as-grown film. Reduction further lowers crystallinity. **b** Atomic-resolution cross-sectional HAADF-STEM micrograph from the reduced SrTiO_3_/Nd_0.8_Sr_0.2_NiO_2_ film showing amorphous (marked as 1) and crystalline regions (marked as 2 and 3). The low-crystalline quality of the film after reduction is clearly visible and agrees with the XRD data. **c** SIMS depth profile of the as-grown Nd_0.8_Sr_0.2_NiO_3_ film indicates a separate surface layer. **d** SIMS depth profile of the reduced Nd_0.8_Sr_0.2_NiO_2_ film with a SrTiO_3_ cap, showing non-negligible but small hydrogen incorporation at the SrTiO_3_ cap/Nd_0.8_Sr_0.2_NiO_2_ interface.

XRD measurements, shown in Fig. 5a, reveal that the crystalline quality of both capped and uncapped films prior to reduction is lower, with broader, lower-intensity film peaks. Importantly, while the (002) Nd_0.8_Sr_0.2_NiO_3_ peak is sharp, the expected perovskite (001) film peak is suppressed; further higher-resolution measurements, shown in Supplementary Fig. 12, resolve the presence of potential Ruddlesden–Popper phase. As before, the topotactic reduction process reduces the *c*-axis lattice parameter, but the peak intensity drops dramatically. Transmission electron micrographs of these samples, such as that shown in Fig. 5b, reveal the segregation of the film into multiple crystalline phases and amorphous-like regions. Thus, unlike the uniform superconducting samples, the aggressive reduction of these films is non-uniform and disordered, resulting in increased mosaicity and a loss of crystalline quality. This is corroborated by electronic transport, as discussed further in the Supplementary Information, which indicates that capped and uncapped films exhibit sharply different resistivities.

As shown in Fig. 5c, which plots SIMS measurements from the as-grown, uncapped Nd_0.8_Sr_0.2_NO_3_ film, the initial transient region shows much higher yields of all ions which may indicate differences in crystallinity near the surface, potentially from the emergence of a polycrystalline scale layer in uncapped samples over time^21^. In the bulk region, the H^−^ and OH^−^ intensities are similar to the substrate.

Despite the significant difference in crystalline quality and reduction conditions, the effects of reduction are similar to our other observations. Figure 5d shows the integrated peak intensities in the reduced SrTiO_3_/Nd_0.8_Sr_0.2_NiO_2_ bilayer. Once again, H^−^ and OH^−^ intensities are elevated at the cap/film interface—though over a broader spatial extent—with almost a 50% increase over the baseline in the substrate. Thus, while the SrTiO_3_ cap appears to trap hydrogen, this enhancement is again far below the orders of magnitude that would be expected for significant hydrogen incorporation, remaining within a factor of two of the substrate values.

### Theoretical Calculations

**Fig. 6:**
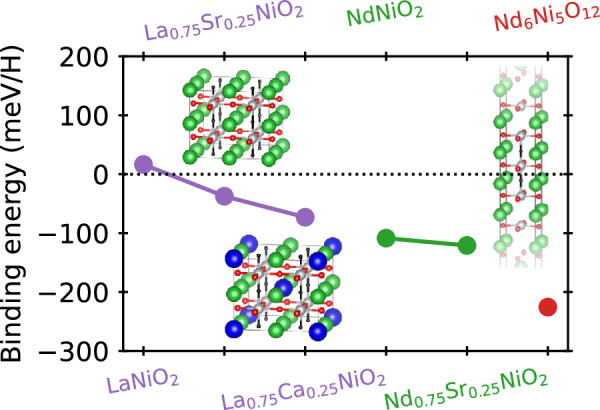
Topotactic-H energetics for superconducting nickelates. Binding energies for hydrogen in superconducting layered nickelates, where a positive (negative) binding energy indicates favoring (disfavoring) H-intercalation. Crystal structures indicate the positions of topotatic-H used for (top) (La,Nd)NiO_2_, (bottom) (La,Nd)_0.75_(Sr,Ca)_0.25_NiO_2_, and (right) Nd_6_Ni_5_O_12_. Solid lines are guides to the eye connecting related materials with the same parent phase e.g., LaNiO_2_ and the associated doped compounds.

To further understand the lack of hydrogen incorporation in the nickelates analyzed above via SIMS (both superconducting and non-superconducting), density-functional theory (DFT)-based calculations were performed to explore the energetics of topotactic hydrogen in infinite-layer *R*NiO_2_ (*R* = rare-earth, both doped and undoped) as well as in the quintuple-layer nickelate Nd_6_Ni_5_O_12_. To investigate whether it is energetically favorable to intercalate hydrogen, we compute the hydrogen binding energy (*E*_*b*_) for the topotactic process as done in previous work^28^:

1 $${E}_{b}=\{E[R{{{{\rm{NiO}}}}}_{2}]+n\times \mu [H]-E[R{{{{\rm{NiO}}}}}_{2}{{{\rm{H}}}}]\}/n,$$

where *E*[*R*NiO_2_] and *E*[*R*NiO_2_H] are the total energies for the infinite-layer *R*NiO_2_ and hydride-oxide *R*NiO_2_H compounds, *μ*[*H*] = *E*[H_2_]/2 is the chemical potential of H, and *n* represents the number of H atoms in the (super)cell. Analogous expressions are used for *R*_0.75_(Sr,Ca)_0.25_NiO_2_ and Nd_6_Ni_5_O_12_. A positive (negative) *E*_*b*_ indicates that the topotatic hydrogen intercalation is favorable (unfavorable). The calculated binding energies are summarized in Fig. 6. We find that the incorporation of H into *R*NiO_2_, *R*_0.75_(Sr,Ca)_0.25_NiO_2_, Nd_6_Ni_5_O_12_ is systematically unfavorable, in agreement with experiments (only for LaNiO_2_ a very small positive E_*b*_ value of approximately 10 meV/H is obtained).

## Discussion

In summary, we searched for hydrogen across a wide range of superconducting and non-superconducting layered nickelate films, with different cation and dopant chemistry, structures, growth methods, reduction conditions, and crystalline quality. Not only did we find no significant concentrations of hydrogen in superconducting films, but we were also unable to use excessive reduction temperature or time to force significant amounts of hydrogen into these structures. These results are consistent with first-principles calculations which show that hydrogen incorporation is energetically unfavorable in both infinite-layer and quintuple-layer nickelates. At most, we observed increased concentrations by a factor of two from the trace amounts already present within the substrates. Furthermore, hydrogen, as hydride or hydroxide ions (H^−^ and OH^−^), was more likely to be found in SrTiO_3_ caps or in the substrates than in the nickelate films themselves. This propensity for hydrogen to appear in higher concentrations in SrTiO_3_ capping layers and SrTiO_3_/nickelate interfaces is interesting in the context of recent work showing the important role such capping layers can play in facilitating the reduction process^21^.

It should be noted that our measurements generally reveal as-grown samples with hydrogen levels at or below the SIMS detection limit prior to reduction, although it is, of course, not possible to completely eliminate hydrogen from any material system. CaH_2_ does appear to introduce hydrogen into the system, as evidenced by changes in both film and substrate levels in as-grown La_0.78_Ca_0.22_NiO_3_ and reduced La_0.78_Ca_0.22_NiO_2_ films, for example. However, topotactic reduction appears unable to introduce hydrogen into these nickelates at the levels near ANiO_2_H_0.25_ (A = La,Sr,Nd,Ca) previously cited as critical doping for superconductivity^27^. At such high levels of incorporated hydrogen, Ding et al. observed H^−^ intensities approximately 40× to 60× the substrate concentration, and approaching a factor of 200× to 600× near the film surface. We find no evidence of such large relative H^−^ intensities in the films studied in this work.

Therefore, although superconductivity is highly sensitive to reduction optimization, this is likely due to the crystalline quality and oxygen stoichiometry, and not hydrogen stoichiometry. Of course, this study does not demonstrate that superconductivity requires the complete absence of incorporated hydrogen. This work instead indicates that many films appear resistant to hydrogen infiltration and that superconductivity may be readily realized at very low hydrogen levels for which no theoretical evidence supports hydrogen-mediated superconductivity.

*Note added:* Recently, independent SIMS experiments performed by Zeng et al.^40^ and Gonzalez et al.^41^ also concluded that extensive hydrogen incorporation is unnecessary for superconductivity in the infinite-layer nickelates, in agreement with this work.

## Methods

### Sample synthesis: thin film deposition and reduction

#### La_1−*x*_(Ca,Sr)_*x*_NiO_2_ films

Thin films, ≈ 6 nm thick, of the infinite-layer nickelates La_0.78_Ca_0.22_NiO_2_ and La_0.8_Sr_0.2_NiO_2_ were grown on SrTiO_3_ (001) substrates using pulsed laser deposition (PLD) and CaH_2_ topotactic reduction^33,34^. SrTiO_3_ (001) substrates were etched with hydrofluoric acid and annealed in air at 900 °C for 90 min before deposition. This is to maximize the TiO_2_ termination which serves to minimize disordered Ruddlesden–Popper type growth. We first grow the perovskite phase using PLD with the following optimal set of parameters: *T*_growth_ = 575 °C, ${{{{\rm{P}}}}}_{{O}_{2}}$ = 150 mTorr (1 Torr = 133.322 Pa), *J* = 2.5 J/cm^2^. Afterwards, the film was post-annealed at growth temperature under the same oxygen partial pressure for 10 min followed by cooling at 8 °C/minute. The topotactic phase transition to the infinite-layer phase was mediated by the substrate strain and performed in the same PLD vacuum chamber with a base pressure of less than 1 × 10^−6^ Torr. The reduction environment was achieved by heating approximately 0.1 g of CaH_2_ powder to obtain a (H_2_ and other species) pressure in the range of approximately 0.1–0.3 Torr. Samples are annealed at 340 °C for 1 h. After reduction, samples were capped with amorphous SrTiO_3_ at room temperature using PLD to protect the surface from reoxidation. Mild oxidation damage to the top nickelate surface can be expected in this process.

#### Nd_6_Ni_5_O_12_ and NdNiO_2_ Films

We use ozone-assisted molecular beam epitaxy (MBE) to synthesize the precursor Nd_6_Ni_5_O_16_/NdGaO_3_ (110) and NdNiO_3_/SrTiO_3_ (001) films in Figs. 3 and 4, respectively. To calibrate the nickel and neodymium elemental fluxes, we synthesize NiO on MgO (001) and Nd_2_O_3_ on yttria-stabilized zirconia (YSZ (111)), then measure the film thickness via X-ray reflectivity. Next, we synthesize NdNiO_3_/LaAlO_3_ (001) and use the *c*-axis lattice constant and film thickness to refine the Nd/Ni ratio and monolayer dose, respectively. Using the optimized neodymium and nickel shutter times from the synthesis of NdNiO_3_/LaAlO_3_, we synthesize the Ruddlesden–Popper nickelates *via* monolayer shuttering. Both NdNiO_3_ and Ruddlesden–Popper nickelates are synthesized at a substrate temperature of 500–600 °C with approximately 1.0 × 10^−6^ Torr distilled ozone (Heeg Vacuum Engineering). The MBE synthesis conditions and calibration scheme are described in Refs. ^10,22^; similar techniques were also used in Refs. ^42,43^.

The perovskite and Ruddlesden-Popper films are reduced to the square-planar phase via CaH_2_ topotactic reduction. The following methods are similar to those used elsewhere^10,20,44^. First, the as-grown films are cut into identical pieces, and the pieces to be reduced are tightly wrapped in aluminum foil (All-Foils) to avoid direct contact between the film and CaH_2_. Each film is then placed in a borosilicate tube (Chemglass Life Sciences) with approximately 0.1 g of CaH_2_ pieces (>92%, Alfa Aesar). The borosilicate tube is pumped down to  <0.5 mTorr, sealed, and then heated for several hours at 290 °C in a convection oven (Heratherm, Thermo Fisher Scientific) with a 10 °C min^−1^ heating rate.

#### Nd_0.8_Sr_0.2_NiO_3_ films

Polycrystalline targets of NdNiO_3_ and Nd_0.8_Sr_0.2_NiO_3_ were prepared by the liquid-mix technique^45,46^. A 10 nm thick Nd_0.8_Sr_0.2_NiO_3_ films were grown on (001) SrTiO_3_ substrates using a Neocera PLD system equipped with an in-situ RHEED (Staib Instruments, Germany). The depositions were conducted using a KrF excimer laser operating at 2 Hz with a fluence of 1.5 J cm^−2^. During the deposition, a dynamic oxygen pressure of 150 mTorr was maintained, and the substrate temperature was 735 °C. The optional 10 nm thick SrTiO_3_ capping layer was grown at the same condition as the film. After the deposition, all samples were in-situ annealed at the deposition temperature in an oxygen atmosphere of 500 Torr for 30 min and subsequently cooled to room temperature at a rate of 15 °C min^−1^.

The as-grown films were sealed in evacuated (approximately 1 mTorr) quartz ampoules with 0.1 g CaH_2_ powder (90%–95%, Thermo Scientific Chemicals). The ampoules were then baked in a muffle furnace at 600 °C for up to 10 h. The temperature ramp rate was fixed at 10 °C min^−1^. Once the ampoules were opened, the reduced films were immediately rinsed in *n*-butanol and isopropanol in an ultrasonic bath for 3 min.

### X-ray diffraction

X-ray diffraction (XRD) measurements were performed at room temperature before and after reduction by each group on commercially available X-ray diffractometers using Cu K*α*_1_ (*λ* = 1.5406 Å) radiation.

### X-ray reflectometry

X-ray reflectometry (XRR) measurements were performed at ambient conditions in a horizontal configuration using a Rigaku SmartLab diffractometer. The incident beam was collimated using the parallel beam slit and an incident slit of 30 μm height to improve *Q*-resolution. The Cu K*α*_1_ wavelength (*λ* = 1.5406 Å) was isolated by using a Ge-(220) × 2 monochromator. The scattered beam was further collimated by a 0.2 mm receiving slit. The data were reduced using the *reductus* web-service^47^ and fit to a slab model using Refl1D^48^.

### Time-of-flight secondary ion mass spectroscopy

Time-of-flight SIMS was performed using an IONTOF IV (Münster, Germany) equipped with a 20 keV ${{{{\rm{Ar}}}}}_{2600\pm 1000}^{+}$ cluster source for sputtering, a 30 keV ${{{{\rm{Bi}}}}}_{3}^{+}$ liquid metal ion source for analysis, and a time-of-flight mass analyzer. Depth profiling was performed in non-interlaced mode with 1 scan of analysis with a lateral resolution of (128 × 128) pixels, 10 scans of sputtering, and at least 0.5 s of charge compensation per cycle, where both the analysis and sputter rasters were kept inside a (500 × 500) μm area. The corresponding ion doses were 1.9 × 10^9^ ions/cm^2^ (0.12 pA) for Bi_3_^+^, and between 2.1 × 10^14^ ions/cm^2^ to 2.6 × 10^14^ ions/cm^2^ (5.1–6.4 nA) per cycle for the cluster source due to day-to-day fluctuations in the beam current. On especially insulating samples or substrates, a small drop of silver paint was used to electrically contact the sample surface to the sample holder for further charge compensation.

For reliable detection of H^−^ ions, contributions from residual gases were minimized by keeping the chamber pressure below 5 × 10^−7^ Pa. Both negative and positive ions were analyzed at separate spots, and the signal rastered over multiple spots was averaged after normalizing for the highest intensity ion unique to the substrate (${{{{\rm{TiO}}}}}_{2}^{-}$ or ${{{{\rm{GaO}}}}}_{2}^{-}$).

Spectra were analyzed using SurfaceLab to define a region of interest, perform mass calibrations, identify peaks with the appropriate compounds, and extract the total integrated peak intensity as a function of sputter time. As many molecular compounds can have similar mass, peak assignments were made carefully, considering factors such as mass offset, isotopic distribution, and similarity in profile shape to other known oxide and hydroxide species.

### Electron microscopy

All cross-sectional STEM specimens were prepared by the standard focused ion beam (FIB) lift-out procedure and imaged in high-angle annular dark-field (HAADF)-STEM configuration. The instrument, processing, and experimental details for specific samples are as follows:

- Nd_6_Ni_5_O_12_. Preparation: Thermo Fisher Scientific Helios G4 UX and FEI Helios 660 FIBs. Imaging: probe-corrected Thermo Fisher Scientific Spectra 300 X-CFEG operating at 300 kV, 19 mrad convergence semi-angle, 33 mrad inner collection angles.
- NdNiO_2_. Preparation: FEI Helios 660 FIBs with final polishing at 5 kV accelerating voltage and 41 pA probe current. Imaging: Thermo Fisher 615 Scientific Titan Themis Z G3 operating at 200 kV, 18.9 mrad convergence semi-angle, and 68 (280) mrad inner (outer) collection angles.
- SrTiO_3_-capped (Nd,Sr)NiO_3−*x*_ and (Nd,Sr)NiO_2_. Preparation: Thermo Fisher Scientific Helios G4 UXe PFIB Dual Beam, with final polishing at 5 kV accelerating voltage. Imaging: Thermo Fisher Scientific Spectra 200 operating at 200 kV, 25 mrad convergence semi-angle, 54 (200) mrad inner (outer) collection angles, and probe current of approximately 20 pA. Energy dispersive X-ray spectroscopy (EDS) chemical mapping was performed on the same instrument with Bruker Dual-X X-ray detectors an electron beam current of approximately 100 pA.

### Computational methods

Density-functional theory (DFT)-based calculations were performed to theoretically explore the energetics of topotactic hydrogen in the infinite-layer nickelate *R*NiO_2_ (*R* = La, Nd, both doped and undoped) as well as in the quintuple-layer nickelate Nd_6_Ni_5_O_12_. For *R*NiO_2_H_*δ*_, *R*_0.75_(Sr,Ca)_0.25_NiO_2_H_*δ*_, and Nd_6_Ni_5_O_12_H_*δ*_ (*δ* = 0, 1) structural relaxations were performed using the VASP code^49–51^ with the the Perdew–Burke–Ernzerhof version of the generalized gradient approximation (GGA-PBE)^52^. For the infinite-layer materials (*R*NiO_2_ and *R*_0.75_(Sr,Ca)_0.25_NiO_2_) up to a 2 × 2 × 2 supercell was used to accommodate the appropriate H content and/or (Sr,Ca)-doping level. We place the topotatic-H at the positions of the (removed) apical oxygens as this was shown to be the most energetically favorable position for H-incorporation from previous works^27–30^. GGA-PBE was chosen as it provides lattice constants in close agreement with experimental data, as shown in Supplementary Note H. A *Γ*-centered 13 × 13 × 15 (9 × 9 × 11) *k*-mesh was used for the 1 × 1 × 1 unit cells (2 × 2 × 2 supercells) with a 0.05 eV Gaussian smearing. For Nd_6_Ni_5_O_12_, a *Γ*-centered 9 × 9 × 9 *k*-mesh with a 0.05 eV Gaussian smearing was used. The size of the plane-wave basis sets was set with a kinetic energy cut-off of 520 eV. For *R* = Nd, we have used a pseudopotential where the Nd(4*f*) electrons are frozen in the core. To compute the chemical potential of hydrogen (*μ*[H]), we optimized an H_2_ dimer in 15^3^ Å^3^ box with energy cutoff set to 325 eV.

## Supplementary information

Supplementary Information

Peer Review File

## Data Availability

The data supporting this study have been deposited in Figshare.

## Code availability

Analysis was performed by open-source Python packages, including NumPy, matplotlib, and SciPy. SIMS data reduction was performed using the commercial software SurfaceLab 7. All density-functional theory calculations were performed with the Vienna Ab-Initio Simulation Package (VASP).
