# Supplementary material for: Extensive hydrogen incorporation is not necessary for superconductivity in topotactically reduced nickelates
Source: Nat Commun. 2024 Aug 27;15:7387. doi: 10.1038/s41467-024-51479-3 (PMC11349950; doi:10.1038/s41467-024-51479-3)
Supplement: Supplementary file 1 — Supplementary Information [file 41467_2024_51479_MOESM1_ESM.pdf]

Supplementary Information for: Extensive hydrogen incorporation is not necessary for superconductivity in topotactically reduced nickelates

Purnima P. Balakrishnan[†,1,*] Dan Ferenc Segedin[2,†] Lin Er Chow[3,†] Paige E. Quarterman[1] Shin Muramoto[4] Mythili Surendran[5,6] Ranjan K. Patel[7] Harrison LaBollita[8] Grace A. Pan[2] Qi Song[2] Yang Zhang[9] Ismail El Baggari[9] Koushik Jagadish[5] Yu-Tsun Shao[5,10] Berit H. Goodge[11,12,13] Lena F. Kourkoutis[11,12] Srimanta Middey[7] Antia S. Botana[8] Jayakanth Ravichandran[5,6,14,‡] A. Ariando[3,§] Julia A. Mundy[2,¶] and Alexander J. Grutter[1,**]

[1] *NIST Center for Neutron Research, National Institute of Standards and Technology, Gaithersburg, MD 20899, USA*
[2] *Department of Physics, Harvard University, Cambridge, MA 02138, USA*
[3] *Department of Physics, Faculty of Science, National University of Singapore, Singapore 117551, Singapore*
[4] *Material Measurement Laboratory, National Institute of Standards and Technology, Gaithersburg, MD 20899, USA*
[5] *Mork Family Department of Chemical Engineering and Materials Science, University of Southern California, Los Angeles, CA 90089, USA*
[6] *Core Center for Excellence in Nano Imaging, University of Southern California, Los Angeles, CA 90089, USA*
[7] *Department of Physics, Indian Institute of Science, Bengaluru, 560012, India*
[8] *Department of Physics, Arizona State University, Tempe, AZ 85287, USA*
[9] *The Rowland Institute at Harvard, Harvard University, Cambridge, MA 02138, USA*
[10] *Core Center for Excellence in Nano Imaging, University of Southern California, 925 Bloom Walk, Los Angeles, CA 90089, USA*
[11] *School of Applied and Engineering Physics, Cornell University, Ithaca, New York 14853,USA*
[12] *Kavli Institute at Cornell for Nanoscale Science, Ithaca, New York 14853, USA*
[13] *Max Planck Institute for Chemical Physics of Solids, 01187 Dresden, Germany*
[14] *Ming Hsieh Department of Electrical and Computer Engineering, University of Southern California, Los Angeles, CA 90089, USA*

(Dated: August 6, 2024)

Supplementary Note 1: Sample Summary

Supplementary Table 1 lists details of all samples referenced in this paper, along with their fabrication details and which figures they correspond to.

Supplementary Note 2: Hydrogen Quantification using SIMS

Although ToF-SIMS is very sensitive to mass and depth, extracting quantitative information (e.g., chemical stoichiometries) can be challenging due to so-called matrix effects where the chemical and electronic environment of the material (matrix) affects the ionization of the sputtered species. First, the choice of the sputtering beam can change the matrix. For example, the use of a Cs^+ sputter beam results in Cs implantation into the film, lowering the work function and dramatically increasing the yield of negative ions [1]. Because different ions can interact differently with the implanted Cs, the ion yield ratios can vary drastically. To minimize potential chemical interactions with the sample, we chose to use an Ar^+ noble gas cluster source for sputtering, which is also volatile and escapes to vacuum after bombardment. The type of analysis beam ($Bi_3{}^+$, $O_2{}^+$, Ga^+, etc.) also has an effect on ionization, and this needs to be specified when comparing quantitative results.

* purnima.balakrishnan@nist.gov
† equal contribution
‡ j.ravichandran@usc.edu
§ phyarian@nus.edu.sg
¶ mundy@fas.harvard.edu
** alexander.grutter@nist.gov

Label	Material	Synthesis	Figures
As-grown (La,Ca)NiO_3	5.4 nm $La_{0.78}Ca_{0.22}NiO_3$ / $SrTiO_3$	1	2b,c,d, Supplementary Figure 6
Superconducting (La,Ca)NiO_2	29 nm $SrTiO_3$ / 4 nm $La_{0.78}Ca_{0.22}NiO_2$ / $SrTiO_3$		2a,b,c,d,e,f, Supplementary Figure 6
As-grown (La,Sr)NiO_3	5.3 nm $La_{0.8}Sr_{0.2}NiO_3$ / $SrTiO_3$		2b, Supplementary Figure 5
Superconducting (La,Sr)NiO_2	6 nm $SrTiO_3$ / 4.2 nm $La_{0.8}Sr_{0.2}NiO_2$ / $SrTiO_3$		2a,b,g,h, Supplementary Figure 3, Supplementary Figure 5
Superconducting $Nd_6Ni_5O_{12}$	100 nm Pt / 10 nm Ti / 23 nm $Nd_6Ni_5O_{12}$ / $NdGaO_3$	2	1c, 3a,b,c, Supplementary Figure 7
As-grown $NdNiO_3$	17.3 nm $NdNiO_3$ / $SrTiO_3$	2	4a,c,d, Supplementary Figure 8, Supplementary Figure 10
Reduced $NdNiO_2$	17.3 nm $NdNiO_2$ / $SrTiO_3$		4a,e,f, Supplementary Figure 2, Supplementary Figure 8, Supplementary Figure 10
Reduced $NdNiO_2$	16 nm $NdNiO_2$ / $SrTiO_3$		4b
As-grown (Nd,Sr)NiO_3	10 nm $Nd_{0.8}Sr_{0.2}NiO_3$ / $SrTiO_3$	3	5a,c,d, Supplementary Figure 11a, Supplementary Figure 9, Supplementary Figure 12
As-grown capped (Nd,Sr)NiO_3	10 nm $SrTiO_3$ / 10 nm $Nd_{0.8}Sr_{0.2}NiO_3$ / $SrTiO_3$		5a, Supplementary Figure 11b, Supplementary Figure 12
Reduced (Nd,Sr)NiO_2	10 nm $Nd_{0.8}Sr_{0.2}NiO_2$ / $SrTiO_3$		5a, Supplementary Figure 12
Reduced capped (Nd,Sr)NiO_2	10 nm $SrTiO_3$ / 10 nm $Nd_{0.8}Sr_{0.2}NiO_2$ / $SrTiO_3$		5b,e,f, Supplementary Figure 9, Supplementary Figure 12, Supplementary Figure 13, Supplementary Figure 14
Mica	$(KF)_2(Al_2O_3)_3(SiO_2)_6(H_2O)$	MTI Corp.	Supplementary Figure 1

Supplementary Table 1. **Table of samples and where they are referenced in this paper.** Grouped samples are pieces split from the same deposited film.

Second, quantification requires matrix-matched standards. Since even small impurities can affect ionization, standards need to be prepared with known ratios of chemical species and exist in a similar chemical environment to that of the films of interest [2]. Relative sensitivity factors can then be calculated to scale the integrated peak areas to atomic concentrations. We first attempt to quantify hydrogen content in our nickelate films by comparing to a mica standard, as used in Ding *et al.* [3]. The chemical formula for the mica (muscovite) crystals we used is $(KF)_2(Al_2O_3)_3(SiO_2)_6(H_2O)$. Peak shapes as well as intensity measurements are shown in Supplementary Figure 1.

To illustrate the importance of matrix-matched standards, we compare the levels of oxygen (O^- and O_2^-) measured in the mica and in the $SrTiO_3$ and $NdGaO_3$ substrates prior to any film reduction. Although they have comparable oxygen number densities, we found that dissimilarities in the ionization efficiencies produced drastically different results (i.e., estimated stoichiometric ratios). A quick calculation revealed that the apparent oxygen concentration was significantly lower in the mica than in the $SrTiO_3$ and $NdGaO_3$ substrates, and this is due to the presence of different elements in the matrix. Similar matrix-related effects are usually observed for all elements of the periodic table, including H, which highlights the difficulty of quantification using SIMS. In addition, hydrogen is present in adventitious carbon contamination at surfaces and interfaces, and requires careful interpretation of the profile data.

Another complication is the effect of the reduction process on the chemical and physical properties of the film, which directly affects the sensitivity factor and the sputtering rate, respectively. A lower sputter rate means that fewer number of elements and molecules are being ejected at any given time, and even though the ionization efficiency may not have changed, the intensity appears to be lower. In our study, this apparent intensity of molecular species within a single nickelate sample varied dramatically as a function of reduction time and conditions, as discussed in further detail in Supplementary Note 5. This renders even samples cleaved from a single growth and reduced differently to not be quantitatively comparable.

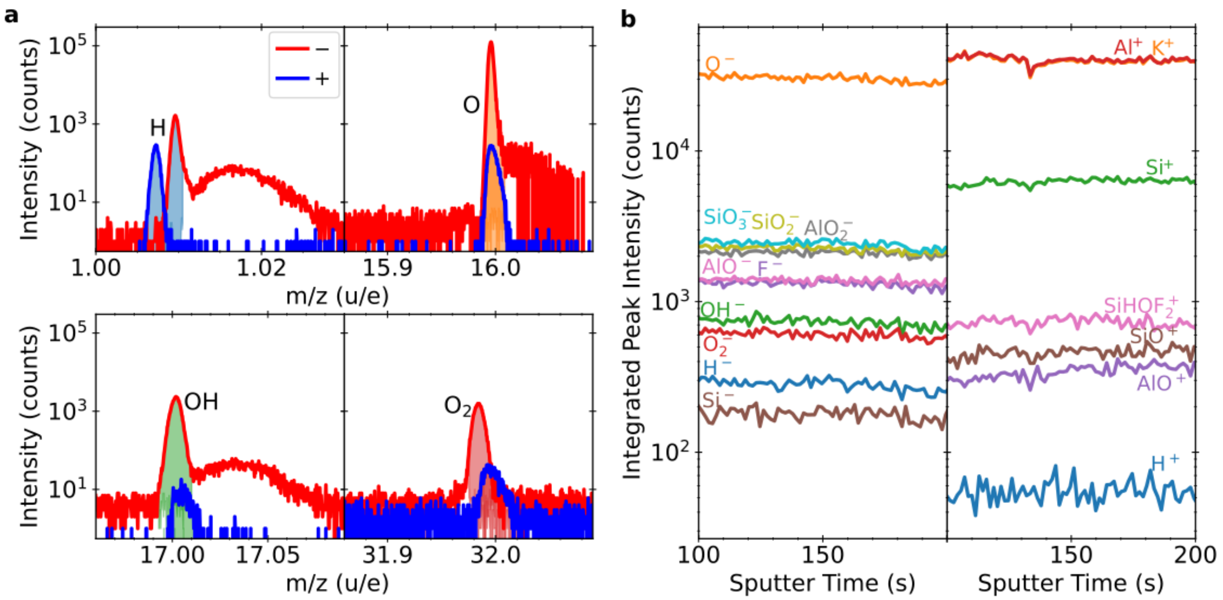

Supplementary Figure 1. **ToF SIMS measurements of mica** a. SIMS showing the spectral peak shapes of $H^{\pm}$, $O^{\pm}$, $OH^{\pm}$, and $O_2^{\pm}$ ions. The broad peaks centered around $m/z = 1.02$ and $m/z = 17.03$ are attributed to charging effects, and were not included for the construction of the depth profile. b. SIMS depth profiles showing the integrated peak intensities as a function of sputtered depth for both elemental and molecular ions from a mica substrate.

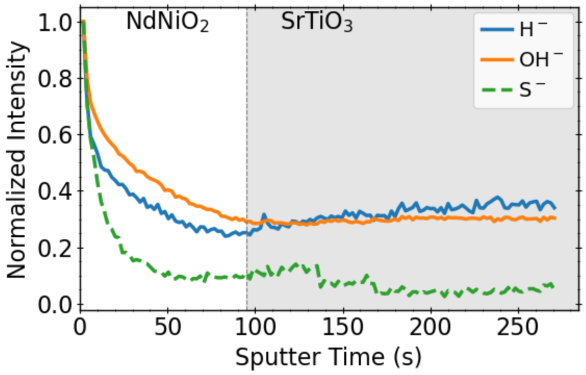

Supplementary Figure 2. **Comparison of ToF-SIMS hydrogen signals in reduced non-superconducting $NdNiO_2$ with surface adsorbate signals.** Intensities have been normalized to the surface value. The slower decay of hydrogen intensities indicate that some of the hydrogen signal is intrinsic to the sample.

Supplementary Note 3: Disentangling Extrinsic and Intrinsic Hydrogen

As mentioned earlier, SIMS can also detect surface adsorbates, and requires a way to distinguish hydrogen intrinsic to the sample from surface contamination. Surface adsorbates are often hydrocarbons, so we can compare carbon peaks such as C_2^-, as well as other contaminant peaks, and see if hydrogen trends with these compounds. The rapid decay in signal at the surface is usually due to these hydrocarbons, and their contribution to the H and OH signals can be subtracted from those inside the film. We can further confidently attribute certain signals to measured hydrogen by isotope analysis. Metal oxides such as Ti will have the same ionization efficiency regardless of the isotope (e.g. $^{48}TiO_2$ vs. $^{49}TiO_2$). Species with isobaric interference, or those that overlap in mass, such as $^{49}TiO_2$ and $^{48}TiO_2H$, can be distinguished by analyzing

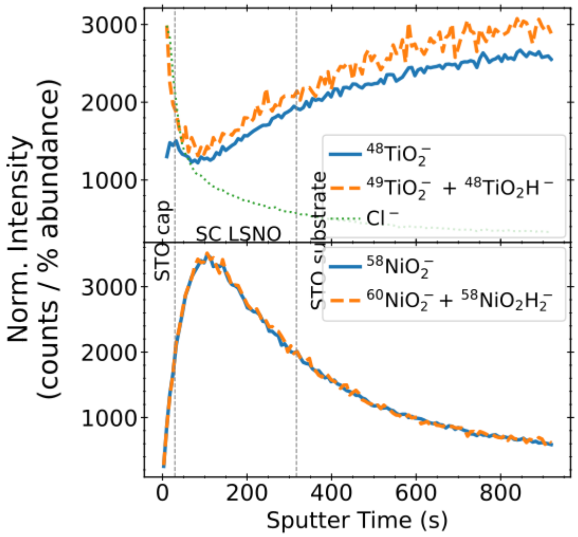

Supplementary Figure 3. **Comparison of the isotopic distributions of Ti and Ni oxides in superconducting $La_{0.8}Sr_{0.2}NiO_2$.** The less abundant isotopic oxides have an overlapping mass with the more abundant isotopic hydroxides. By scaling the depth profiles by the natural isotopic abundance, we can separate out these contributions. In this case, there is a contribution from the Ti hydroxide, but no evidence of Ni hydroxide. The sharp decay in peak intensity for $^{48}TiO_2H^-$ at the surface indicates that the Ti oxide of the $SrTiO_3$ cap has adsorbed some hydrogen, but that has not affected the nickelate film.

the natural isotopic distribution in the mass spectrum or profile shape, as shown in Supplementary Figure 3. In this figure, the less abundant $^{49}TiO_2{}^-$ has an overlapping mass with $^{48}TiO_2H^-$ at $m/z = 64.945$ that cannot be resolved. However, by constructing the depth profile and seeing their profile shapes, it is possible to identify which molecule the peak corresponds to. In this case, the main contribution to the peak is from the Ti hydroxide. Similarly for Ni oxides, comparison of the profiles show that $^{60}NiO_2^-$ is the likely assignment for this peak instead of the hydroxide. The sharp decay in peak intensity for $^{48}TiO_2H^-$ at the surface indicates that the Ti oxide of the $SrTiO_3$ cap has adsorbed some hydrogen, but that has not affected the nickelate film.

Supplementary Note 4: Previous detection of hydrogen using SIMS

In order to place our observations into context, it is helpful to consider previous studies which used the ToF-SIMS instrument employed in this study to examine oxide films with large degrees of hydrogen incorporation, such as humidity-treated GdO_xH_y, $LaMnO_3$ membranes exposed to water, and CaH_2-reduced $BaZrO_3$ [4–6]. In Supplementary Figure 4, we show reproduced H^- intensity curves from a $LaMnO_3$ freestanding membrane with incorporated water due to the dissolution of $Sr_3Al_2O_6$ during processing [5]. Also shown is a film of GdO_xH_y which is very near the $Gd(OH)_3$ composition [4]. In these systems, we found enhancements of the H^+, H^-, and OH^- intensities which were 1 to 3 orders of magnitude above the substrate background, and were able to establish the high hydrogen concentrations based on the effects on the material properties. This prior experience provides reasonable upper and lower boundaries for expected effects and demonstrates our sensitivity to intrinsic hydrogen. Interestingly, the results from Ding *et al.* fell between these limits. We conclude therefore that hydrogen would readily have been detected in our measurements of layered square-planar nickelate samples if it were present at previously reported levels.

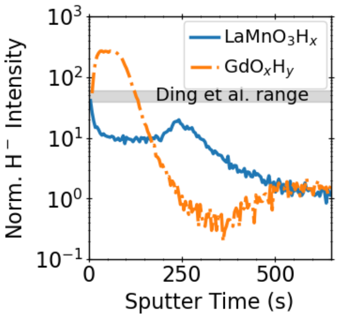

Supplementary Figure 4. **Incorporated H-sensitivity baseline from previous ToF-SIMS measurements.** Examples of previously measured and published SIMS depth profiles for the H^- ion in the hydrogen containing oxides $LaMnO_3H_x$ and GdO_xH_y [4, 5], normalized to 1 in the substrate.

Supplementary Note 5: Extended SIMS Characterization

For each sample, we have recalibrated the SIMS depth profiles by scaling all intensities to an appropriate steady-state ion in the substrate to compare between sample types. This also ensures that small fluctuations in primary ion current between replicate runs can be accounted for. Hydrogen compounds, having much lower intensity, have been scaled as noted for visualization clarity. These are shown in Supplementary Figure 5, Supplementary Figure 6, Supplementary Figure 7, Supplementary Figure 8, and Supplementary Figure 9.

The interface position of the layers is defined as the sputter time when the characteristic signal reaches 50 % of its maximum. The characteristic peaks used were TiO_2^- in the $SrTiO_3$, NiO^- in the nickelate film, and GaO_2^- in the $NdGaO_3$. The interface width is defined as the time or depth required for the intensity to change from 84 % to 16 % of the maximum (or vice versa), and its sharpness is dependent on several factors such as surface roughness, difference in density between the two layers, and redeposition of the sputtered neutrals, in decreasing order of influence. Normally, the sharpest interface is obtained for lower-mass species, and when transitioning from a lower to a higher density film. On the other hand, we observed long NiO_2^- tails in the $SrTiO_3$ substrates, which may likely be attributed to cascade mixing effects induced by the analysis beam knocking atoms deeper into the film[7]. These effects may also account from some of the TiO_2^- signal observed in films capped by $SrTiO_3$, although the nickelate films are denser than the $SrTiO_3$ caps and these films are thin enough that the TiO_2^- is not expected to decay to zero.

We observed that the sputter rate is faster for lower crystallinity layers, including amorphous $SrTiO_3$ caps, as well as reduced films which are lower in crystallinity. In fact, the aggressively reduced $Nd_{0.8}Sr_{0.2}NiO_2$ without protection of the $SrTiO_3$ cap, which exhibited significant crystalline mosaicity with multiple crystalline and amorphous phases, was sputtered through so quickly that no depth profile could be obtained. In general, the $SrTiO_3$ substrate is expected to sputter more quickly than the higher-density films, although film quality also plays a role.

When discussing SIMS "resolution", the most important question is not the interface width but rather our ability to resolve the individual layers and identify their contributions to the signal. This is clearly achieved even in the thinnest ≈ 5 nm thick $La_{0.78}Ca_{0.22}NiO_2$ and $La_{0.8}Sr_{0.2}NiO_2$ superconducting layers, with clear onset and decay of the NiO_2^- signals which yield estimates of the $\pm 1\sigma$ interface widths of approximately 5 nm - 6 nm. We may therefore be confident that the vast majority of the signal in the regions designed "film" originates in the nickelate film, allowing the hydrogen content to be probed.

Thus, SIMS was able to reveal important differences in chemical composition between the as-grown and superconducting samples. As can be seen most clearly in Supplementary Figure 5, CaH_2 reduction preferentially removes oxygen from the nickelate layers. In the as-grown $La_{0.8}Sr_{0.2}NiO_3$ film, the O_2^- signal decreases dramatically at the nickelate/substrate interface, but this decrease is not present after reduction, indicating a lower oxygen content in the nickelate film. Comparison of the as-grown and reduced films (Supplementary Figure 5, Supplementary Figure 6, Supplementary Figure 8, and Supplementary Figure 9) reveals a dramatic decrease in the measured NiO_2^- intensity and NiO_2^-:NiO^- ratio. As the measured intensities of ions is dependent on their oxidation state [8, 9], this change is likely due to the change in the valence state of Ni upon reduction.

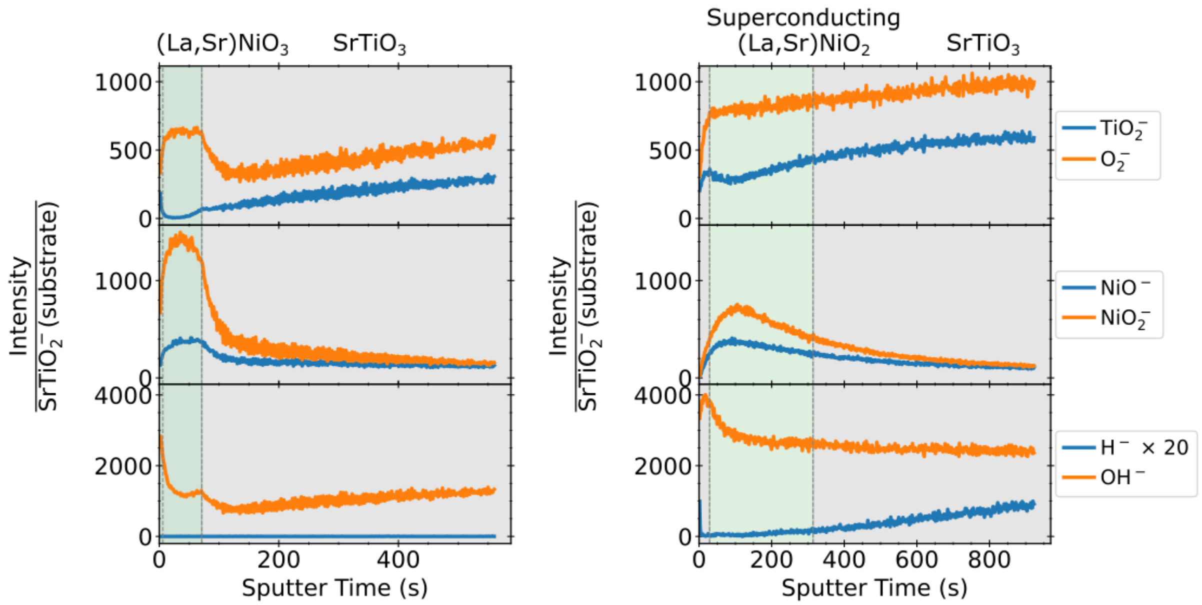

Supplementary Figure 5. **ToF-SIMS depth profiles for the as-grown and superconducting $La_{0.8}Sr_{0.2}NiO_{3\rightarrow 2}$ films**, including those shown in Figure 2. Intensities are normalized to the steady-state level of $SrTiO_2^-$ in the substrate.

Finally, though we see no evidence for extensive hydrogen incorporation in any of these samples, the hydrogen signals which are measured are primarily present as OH^-, and secondarily H^-. The H^+ peak is orders of magnitude smaller, and in many cases undetectable.

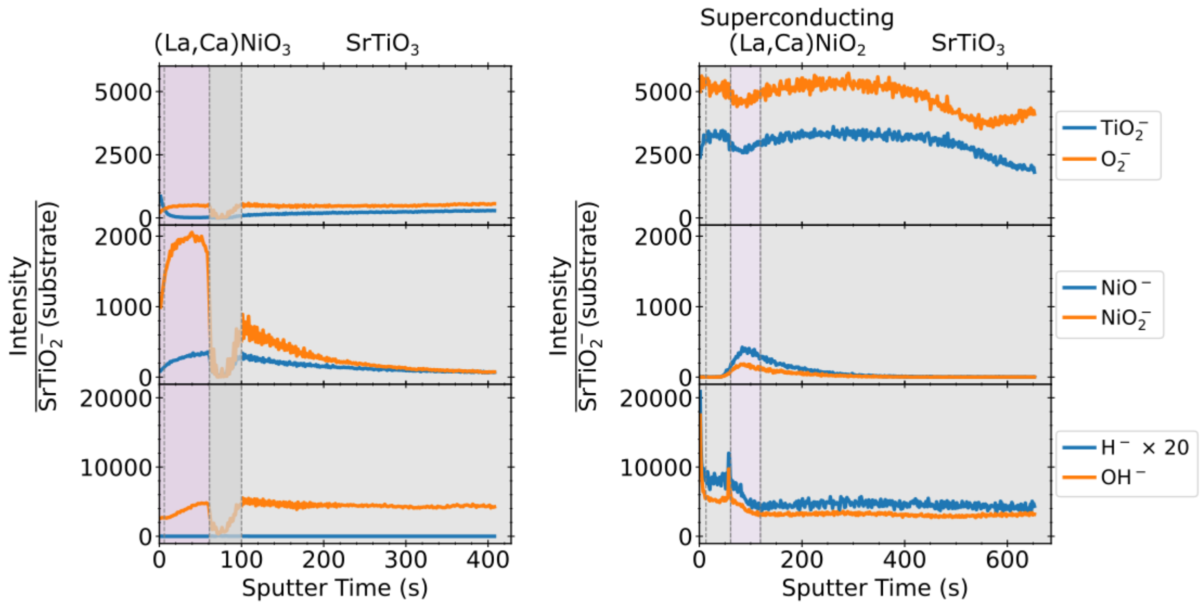

Supplementary Figure 6. **ToF-SIMS depth profiles for the as-grown and superconducting $La_{0.78}Ca_{0.22}NiO_{3\to2}$ films**, including those shown in Figure 2. The greyed out region at the surface of the $SrTiO_3$ substrate of the as-grown film is a measurement artifact due to charging, which decreased the total measured signal.

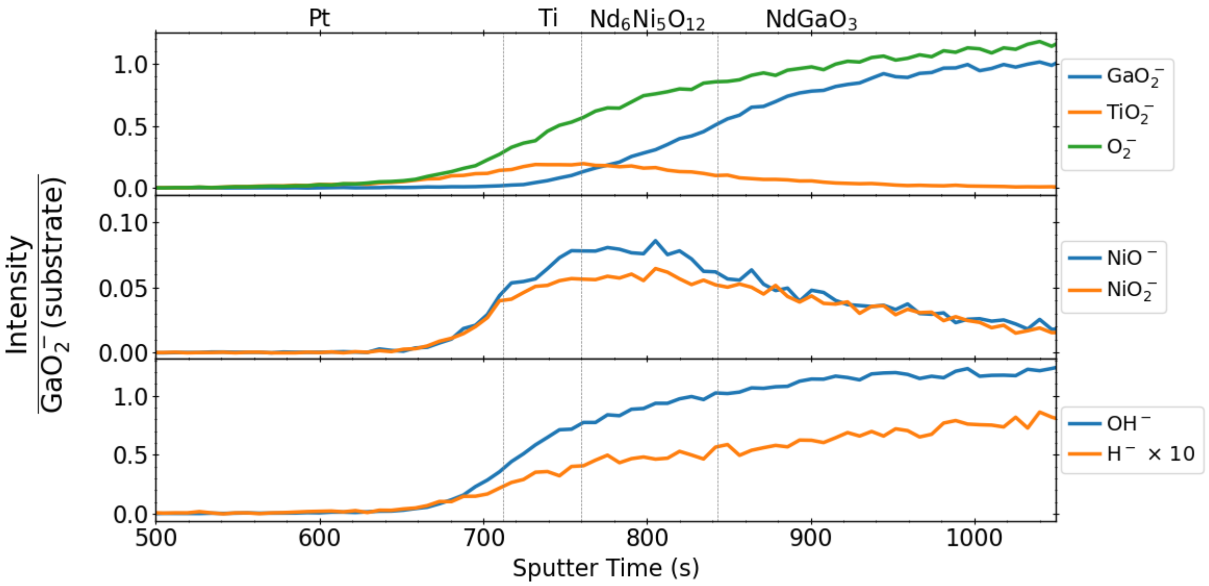

Supplementary Figure 7. **ToF-SIMS depth profile for the $Nd_6Ni_5O_{12}$ film shown in Figure 3.** Intensities are normalized to the steady-state GaO_2^- in the $NdGaO_3$ substrate.

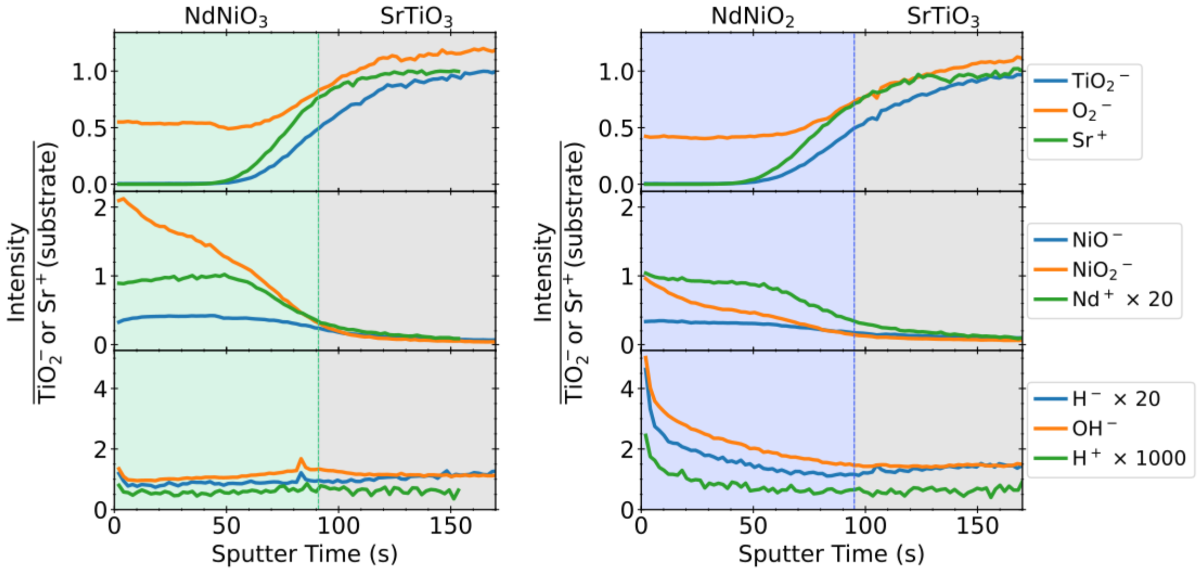

Supplementary Figure 8. **ToF-SIMS depth profiles for the $NdNiO_3$ and non-superconducting $NdNiO_2$** films shown in Figure 4. Intensities are normalized to steady-state TiO_2^- or Sr^+ in the $SrTiO_3$ substrate.

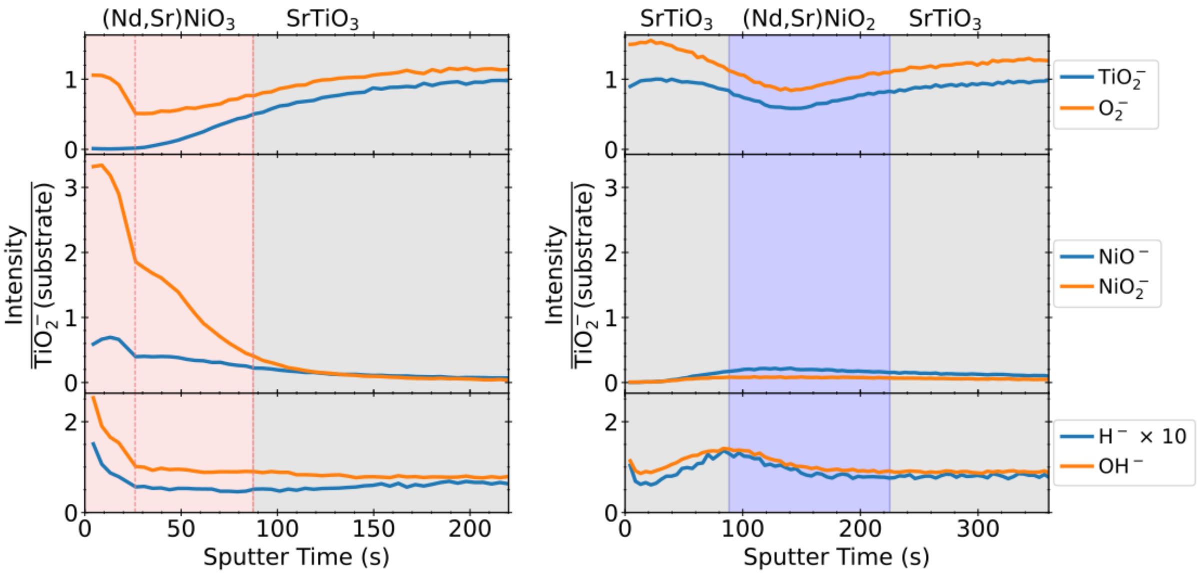

Supplementary Figure 9. **ToF-SIMS depth profiles for the $Nd_{0.8}Sr_{0.2}NiO_3$ and $SrTiO_3/Nd_{0.8}Sr_{0.2}NiO_2$ shown in Figure 5.** Intensities are normalized to steady-state TiO_2^- in the substrate. The surface of the uncapped, as-grown film is likely different due to aging.

Supplementary Note 6: Electrical characterization of non-superconducting films

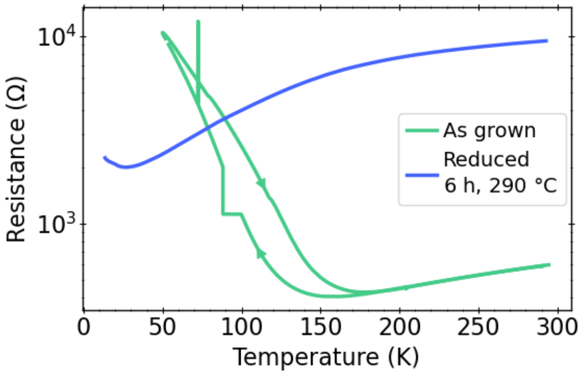

Supplementary Figure 10. **Transport measurements of $NdNiO_{3\rightarrow2}$.** Two-point resistance measurements of as-grown and reduced $NdNiO_{3\rightarrow2}$ shown in Figure 4.

Supplementary Figure 10 shows resistance vs. temperature for the non-superconducting, undoped $NdNiO_3$/$SrTiO_3$ shown in Figure 4. These electrical transport measurements were taken using a home-built electrical dipstick probe compatible with a helium dewar. Indium contacts were soldered on the four corners of each sample in a van der Pauw (VDP) configuration. AC transport measurements were taken at 17.777 Hz using an SR-830 lock-in amplifier. The voltage and current were measured simultaneously to determine the resistance. Note that values shown are the measured resistance of a single VDP channel of these 5 mm×10 mm samples, not sheet resistance or resistivity.

The original as-grown film shows the hysteretic metal-insulator transition expected for a stoichiometric $NdNiO_3$ film, while the reduced infinite-layer film exhibits the metallic behavior expected for undoped $NdNiO_2$.

$Nd_{0.8}Sr_{0.2}NiO_2$ films were insulating enough that two-point resistances were measured only at room temperature. As-grown, without significant growth optimization, uncapped films started with resistances between 500 kΩ to 700 kΩ, while capped films started with resistances between 6 kΩ to 8 kΩ. After reduction, the resistance of uncapped films actually increased and was not measurable using a standard multimeter, while capped films had a lower resistance.

Supplementary Note 7: Further structural characterization

Supplementary Figure 11 shows the RHEED pattern of specular (0 0) and off-specular (0 1), (0 -1) spots from PLD-deposited $Nd_{0.8}Sr_{0.2}NiO_3$ films, recorded after cooling the samples to room temperature. The clear streaky pattern confirms the excellent surface morphology of the films immediately after the deposition.

Supplementary Figure 12 shows full XRD characterization of the capped and uncapped $Nd_{0.8}Sr_{0.2}NiO_{3\rightarrow2}$ films. Complementary XRD measurements were taken using two different XRD configurations. The top scans

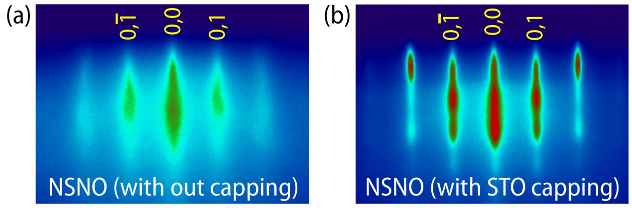

Supplementary Figure 11. **RHEED image of the $Nd_{0.8}Sr_{0.2}NiO_3$ (NSNO) films** (a) with out and (b) with $SrTiO_3$ (STO) capping layer after cooling the samples to room temperature.

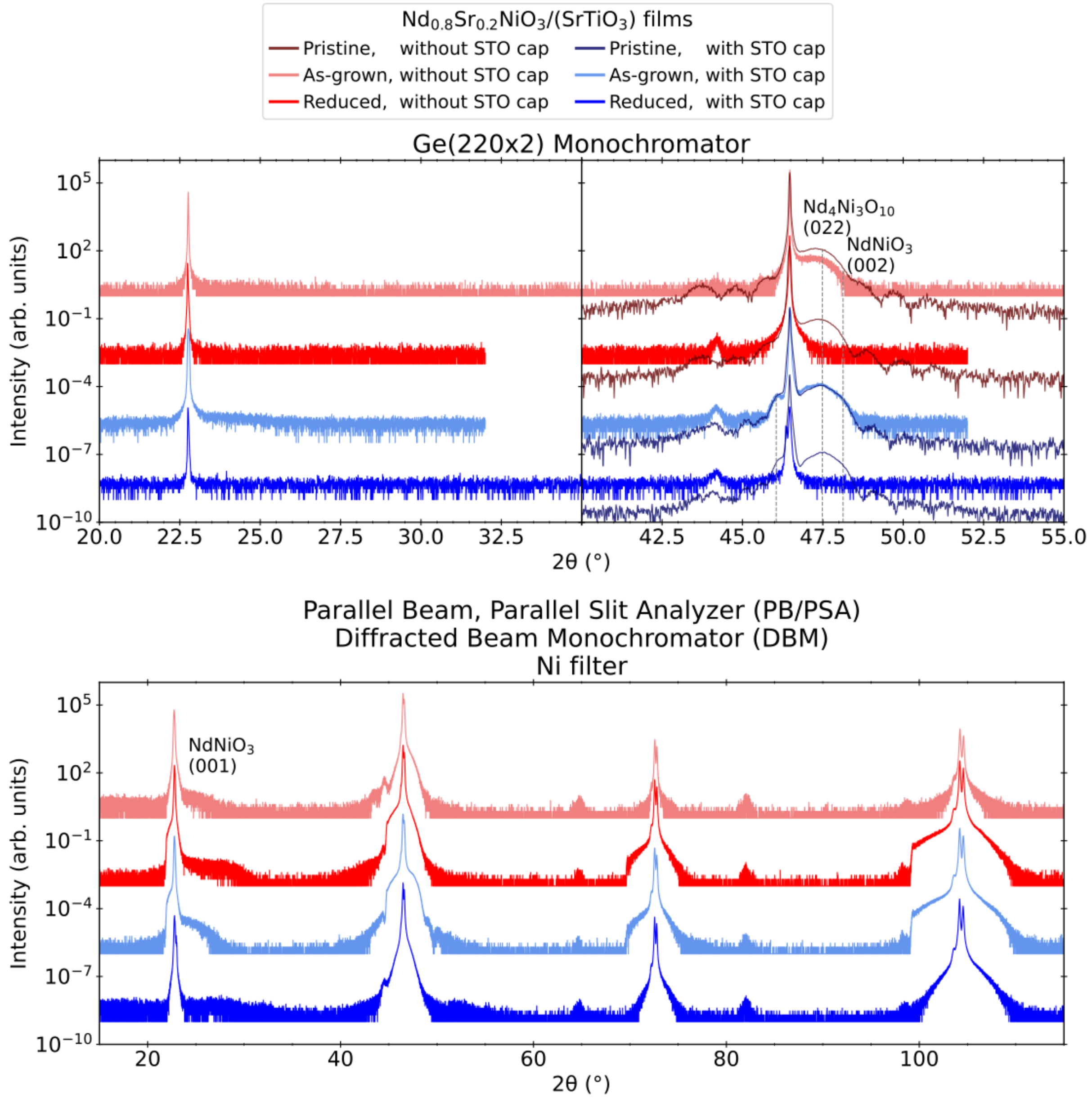

Supplementary Figure 12. **Additional X-ray characterization.** Full XRD scans of samples in Figure 5a taken using two instrument configurations. On top are measurements taken using a lower-intensity, high-resolution mode, while on bottom are the same scans taken in a high-intensity, low-resolution mode to survey for impurity phases.

were measured in a high-resolution mode, using a Ge (220)×2 monochromator to select Cu Kα_1 radiation on the incident side. The Diffracted Beam Monochromator assembly was installed on the receiving side containing a Ni filter, Ge (400)×2 analyzer, and 0.5° PSA. The dark lines represent measurements performed immediately after film deposition (pristine state), while measurements performed on the same films prior to ToF-SIMS are colored as labeled, either as-grown or reduced. The high-resolution measurements allow us to measure the as-grown (002) film peak at 47.485°, and confirm the much lower intensity of the corresponding (001) peak. We note that some slight degradation of the uncapped film occurs over time, consistent with the slightly different surface region seen in ToF-SIMS (Supplementary Figure 9. However, in this measurement mode, the peaks of both reduced films are so low in intensity that they cannot be seen at all. The bottom scans were measured in a high-intensity mode, using a parallel beam (PB) selection slit, and a parallel slit

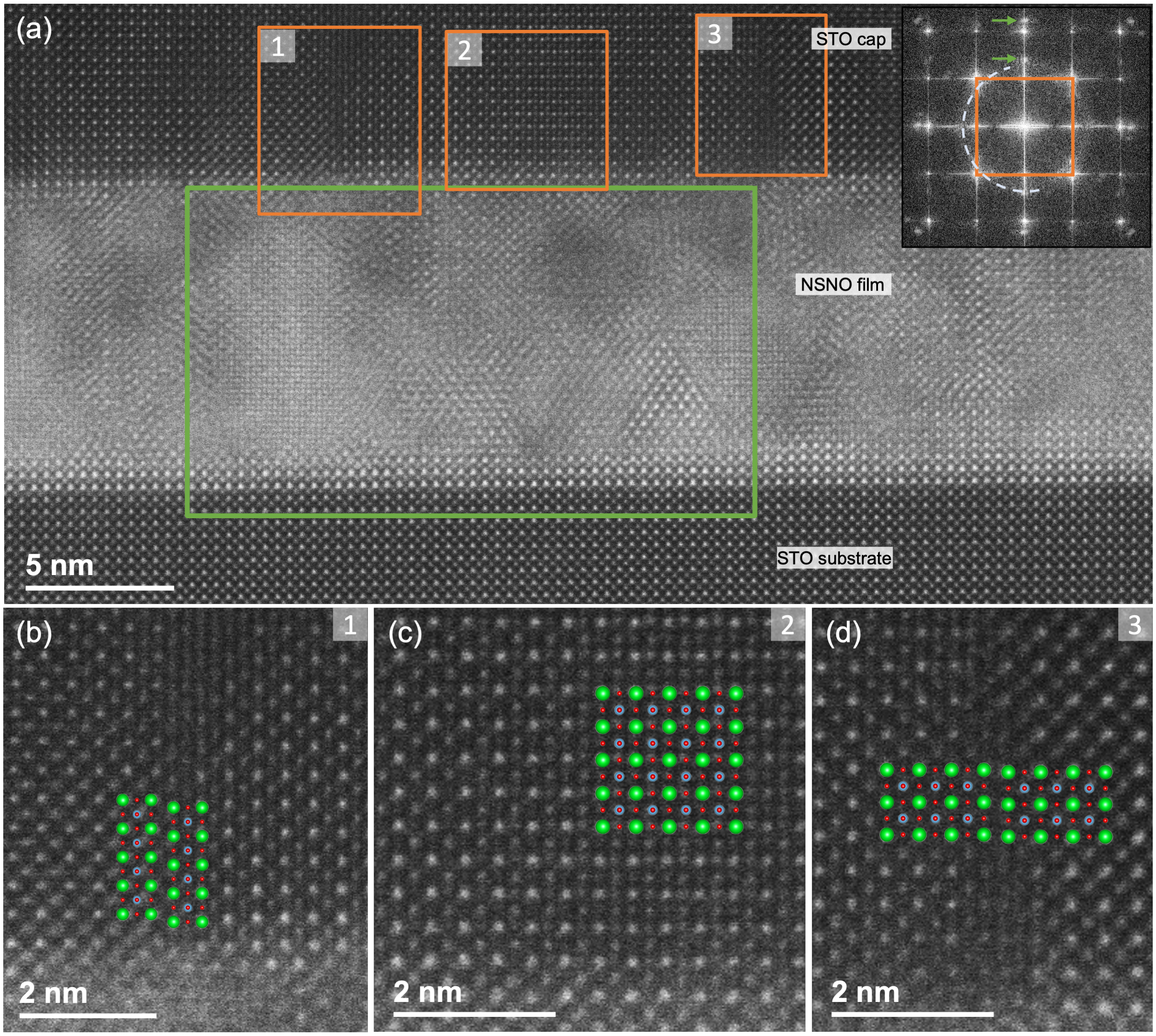

Supplementary Figure 13. **Extended electron microscopy characterization.** (a) The larger field of view of the image shown in Figure 5b, showing the presence of defects in the STO capping layer as well. The inset shows the FFT pattern of the image. The spots corresponding to the cubic STO substrate (and cap) is denoted by the square, the extra spots and the diffused ring indicate the presence of additional crystalline and amorphous regions in the films, as respectively shown by the arrows and dashed semi-circle. (b-d) Magnified regions of the $SrTiO_3$ capping layer showing the presence of various forms of defects, with a schematic structural model overlaid (Sr = green, Ti = blue, O = red spheres). (b) Region 1 shows a Ruddlesden-Popper-like phase at the nickelate-cap interface. (c) Region 2 shows strong signal from the oxygen columns, which cannot be only due to O as low Z elements cannot to scatter to the high collection angle used in this experiment. (d) Region 3 shows the planes in the $SrTiO_3$ cap are highly distorted.

analyzer (PSA). Cu Kα lines were selected using a Ni filter. These measurements allow observation of a (001)-type film peak which shifts to larger angle with reduction.

The crystalline quality and nature of defects in the reduced $SrTiO_3$-capped $Nd_{0.8}Sr_{0.2}NiO_2$ film is studied by cross-sectional HAADF-STEM studies and is observed to be highly defective as shown in Figure 5b. Mosaicity is observed in the (Nd,Sr)NiO_2 layer, appearing as a mixture of amorphous-like and crystalline domains, marked by the circles in Figure 5b. Particularly, two different atomic arrangements deviating from the projected [100] perovskite structure is observed in the crystalline regions marked as 2 and 3 in Figure 5b. Similar defects are present uniformly throughout the observed thin region (~5 µm) of the FIB foil.

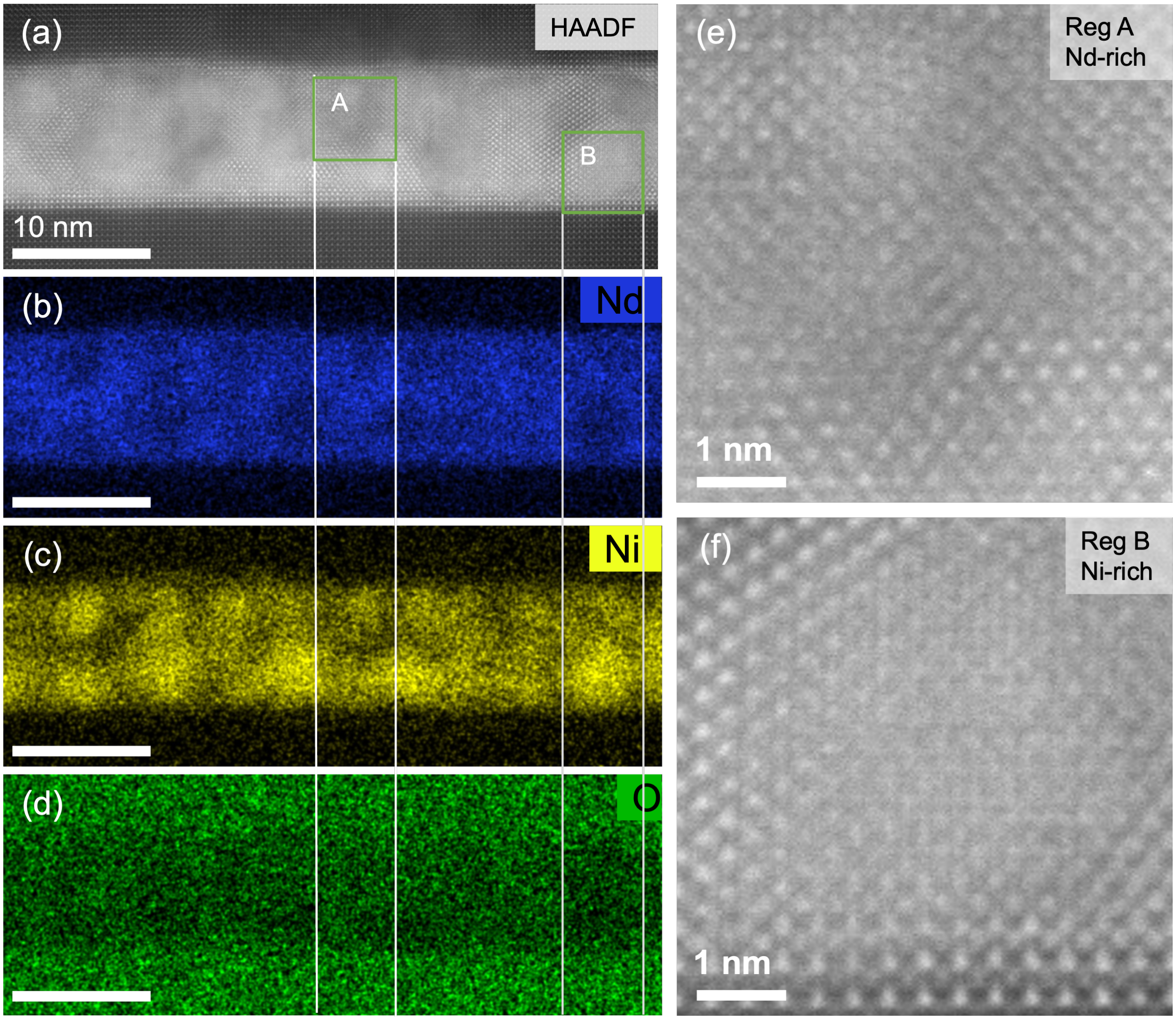

Supplementary Figure 14. **Extended electron microscopy characterization.**(a-d) HAADF-STEM image and the corresponding EDS elemental maps obtained from the reduced $SrTiO_3$-capped (Nd,Sr)NiO_2 film. A clear segregation of Ni and Nd is observed in the nickelate film. (e, f) The amorphous-like and crystalline domains correspond to the Nd-rich and Ni-rich region, respectively.

The Fast Fourier Transform (FFT) of the atomic resolution HAADF-STEM image is shown in the inset of Supplementary Figure 13a. The sharp spots with the 4-fold symmetry corresponds to the $SrTiO_3$ perovskite structure along the [100] orientation. The FFT also shows the presence of additional spots corresponding to periodicities deviating from the STO structure (marked by the green arrows in inset of Supplementary Figure 13a, indicating the occurrence of a phase transformation, most likely during the reduction process. The presence of weak diffused rings corresponds to the presence of the amorphous regions (marked by the dashed circle in inset of Supplementary Figure 13a. The $SrTiO_3$ capping layer is also observed to consist of defects as shown in Supplementary Figure 13b-d.

Further, the chemical nature of the film was studied using STEM-EDS mapping. The elemental maps show chemical segregation in the reduced $SrTiO_3/Nd_{0.8}Sr_{0.2}NiO_2$ film in the form of Ni-rich and Nd-rich regions and shown in Supplementary Figure 14a-d. By correlating the chemical maps with the high-resolution HAADF image, it is observed that the Nd-rich regions correspond to the amorphous-like regions, while the Ni-rich region correspond to the crystalline regions. Finally, Sr was not observed to be concentrated in this film. The STEM results thus provide a direct proof for the crystalline and chemical disorder in the

$Nd_{0.8}Sr_{0.2}NiO_2$ film after the aggressive reduction process.

Supplementary Note 8: Additional DFT data

We provide here the structural data obtained from DFT-based structural relaxations for two choices of exchange-correlation functional: GGA-PBE and GGA-PBEsol. The optimized lattice constants for all the nickelates considered in the main text within these two functionals are summarized in Supplementary Figure 15. We find good agreement with the experimental lattice constants for the undoped infinite-layer compounds (R = La, Nd) is obtained with the GGA-PBE functional, which serves as a benchmark for our DFT-optimized lattice constants (a_{exp}= 3.92 Å, c_{exp}= 3.28 Å for $NdNiO_2$ and a_{exp}=3.96 Å, c_{exp}= 3.37 Å for $LaNiO_2$ [10, 11]). Previous works have used PBEsol to obtain H-binding energies in infinite-layer nickelates [12, 13], but we find that the GGA-PBEsol functional gives rise to optimized lattice constants that are quite far from the experimental values.

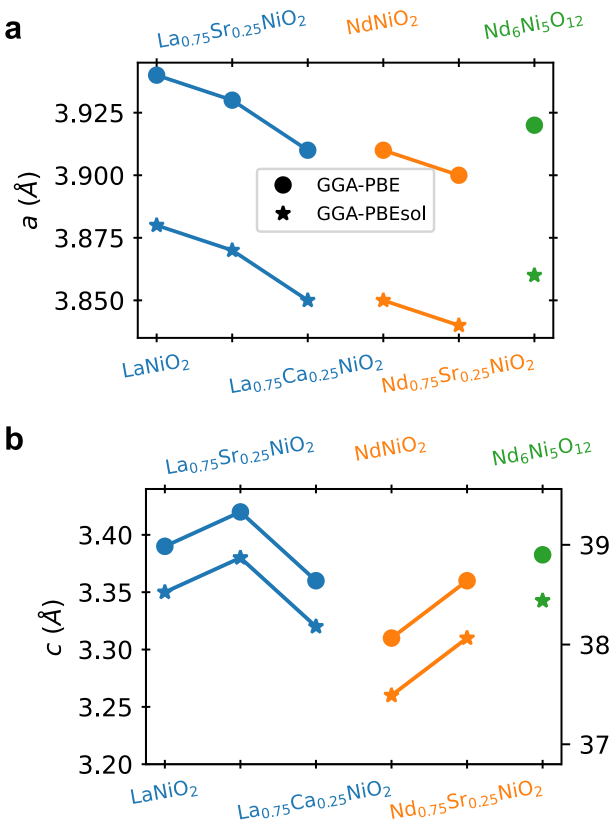

Supplementary Figure 15. **Structural parameters from the DFT-optimized structures.** (a) In-plane lattice constants a. (b) Out-of-plane lattice constants c (right axis corresponds to $Nd_6Ni_5O_{12}$).

Supplementary References

[1] Wehbe, N., Pireaux, J.-J. & Houssiau, L. Xps evidence for negative ion formation in sims depth profiling of organic material with cesium. *The Journal of Physical Chemistry C* **118**, 26613–26620 (2014).

[2] Stevie, F. & Griffis, D. Quantification in dynamic sims: current status and future needs. *Applied Surface Science* **255**, 1364–1367 (2008).

[3] Ding, X. *et al.* Critical role of hydrogen for superconductivity in nickelates. *Nature* **615**, 50–55 (2023). URL https://doi.org/10.1038/s41586-022-05657-2.

[4] Sheffels, S. *et al.* Insight on hydrogen injection and GdOx/Co interface chemistry from in operando neutron reflectometry and secondary ion mass spectrometry. *Applied Physics Letters* **122** (2023). URL https://doi.org/10.1063/5.0128835.

[5] Lu, Q. *et al.* Engineering magnetic anisotropy and emergent multidirectional soft ferromagnetism in ultrathin freestanding $LaMnO_3$ films. *ACS Nano* **16**, 7580–7588 (2022). URL https://doi.org/10.1021/acsnano.1c11065.

[6] Orvis, T. *et al.* Electron doping $BaZrO_3$ via topochemical reduction. *ACS applied materials & interfaces* **11**, 21720–21726 (2019). URL https://doi.org/10.1021/acsami.9b06370.

[7] Magee, C. & Honig, R. Depth profiling by sims—depth resolution, dynamic range and sensitivity. *Surface and Interface Analysis* **4**, 35–41 (1982).

[8] Swart, H. C., Nagpure, I. M., Ntwaeaborwa, O. M., Fisher, G. L. & Terblans, J. J. Identification of Eu oxidation states in a doped $Sr_5(PO_4)_3F$ phosphor by tof-sims imaging. *Opt. Express* **20**, 17119–17125 (2012). URL https://opg.optica.org/oe/abstract.cfm?URI=oe-20-15-17119.

[9] Seed Ahmed, H., Swart, H. & Kroon, R. Investigating the capability of ToF-SIMS to determine the oxidation state of Ce. *Physica B: Condensed Matter* **535**, 124–127 (2018). URL https://www.sciencedirect.com/science/article/pii/S0921452617303824. 7th South African Conference on Photonic Materials SACPM 2017.

[10] Hayward, M. A., Green, M. A., Rosseinsky, M. J. & Sloan, J. Sodium hydride as a powerful reducing agent for topotactic oxide deintercalation: synthesis and characterization of the nickel(i) oxide $LaNiO_2$. *Journal of the American Chemical Society* **121**, 8843–8854 (1999). URL https://doi.org/10.1021/ja991573i.

[11] Hayward, M. & Rosseinsky, M. Synthesis of the infinite layer ni(i) phase $NdNiO_{2+x}$ by low temperature reduction of $NdNiO_3$ with sodium hydride. *Solid State Sciences* **5**, 839–850 (2003). URL https://www.sciencedirect.com/science/article/pii/S1293255803001110. International Conference on Inorganic Materials 2002.

[12] Si, L. *et al.* Topotactic hydrogen in nickelate superconductors and akin infinite-layer oxides ABO_2. *Phys. Rev. Lett.* **124**, 166402 (2020). URL https://link.aps.org/doi/10.1103/PhysRevLett.124.166402.

[13] Si, L., Worm, P., Chen, D. & Held, K. Topotactic hydrogen forms chains in $AB\mathrm{O}_2$ nickelate superconductors. *Physical Review B* **107**, 49–51 (2023). URL https://doi.org/10.1103/PhysRevB.107.165116.
